# Supplementary figures and images for: Differential modification of the C-terminal tails of different α-tubulins and their importance for microtubule function in vivo
Source: eLife. 2023 Jun 22;12:e87125. doi: 10.7554/eLife.87125 (PMC10335831; doi:10.7554/eLife.87125)

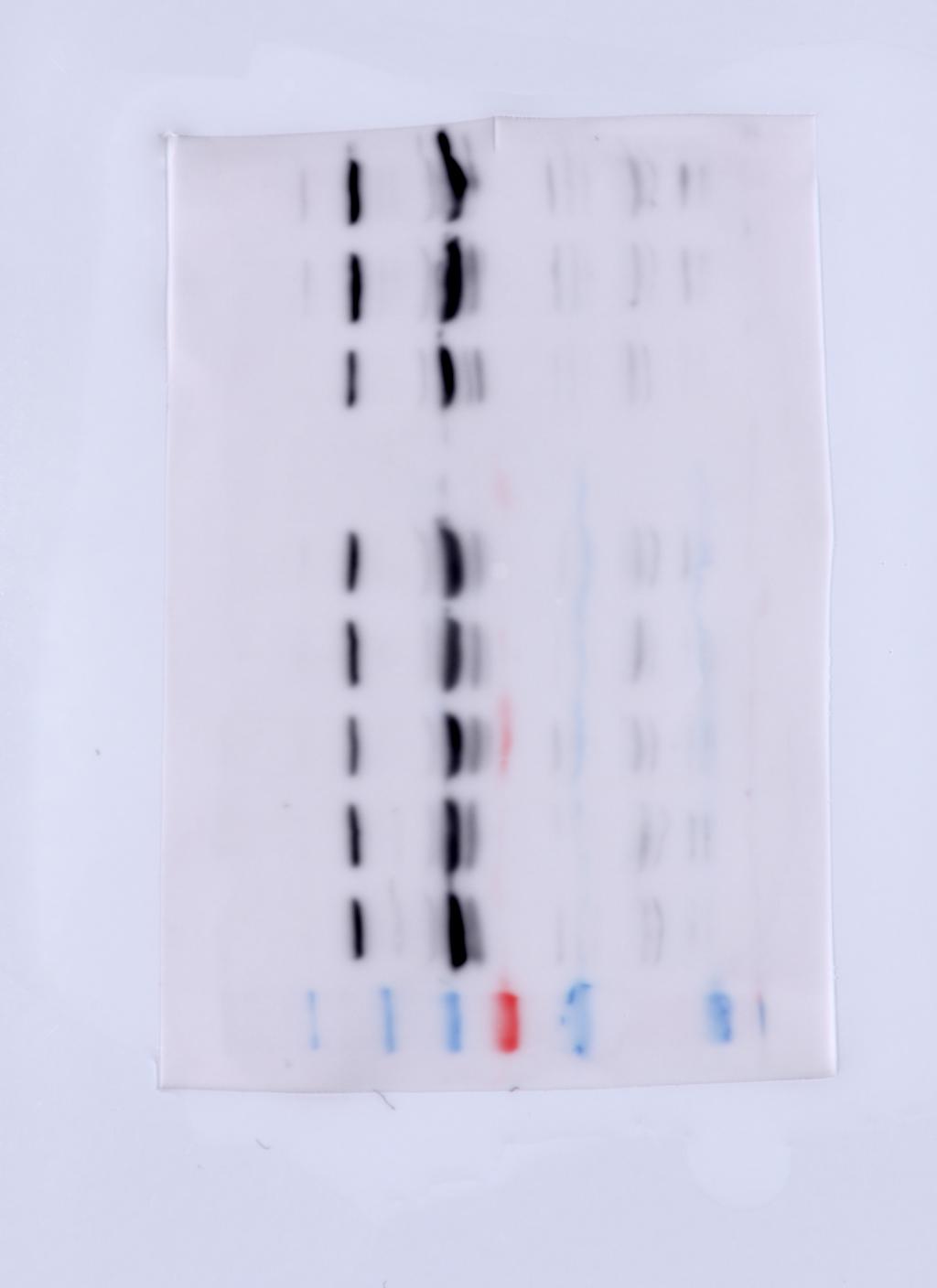

Supplement: Figure 2—source data 1. [file elife-87125-fig2-data1.zip › Figure 2-source data 1/Fig.2_sourceData 1_(2).jpg]

SourceData\_FIG. 2B

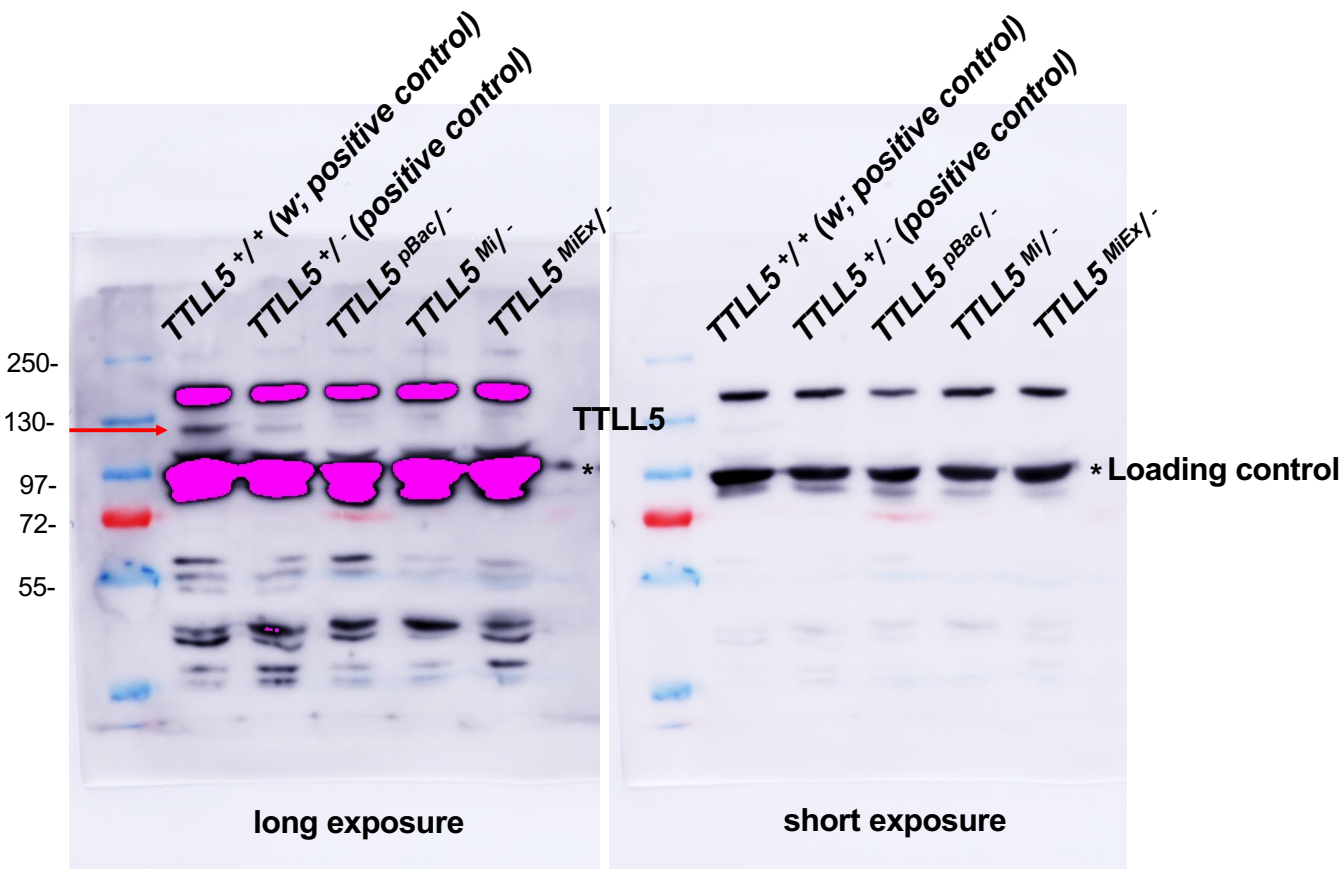

Supplement: Figure 2—source data 1. [file elife-87125-fig2-data1.zip › Figure 2-source data 1/Fig. 2_SourceData 1.pdf]

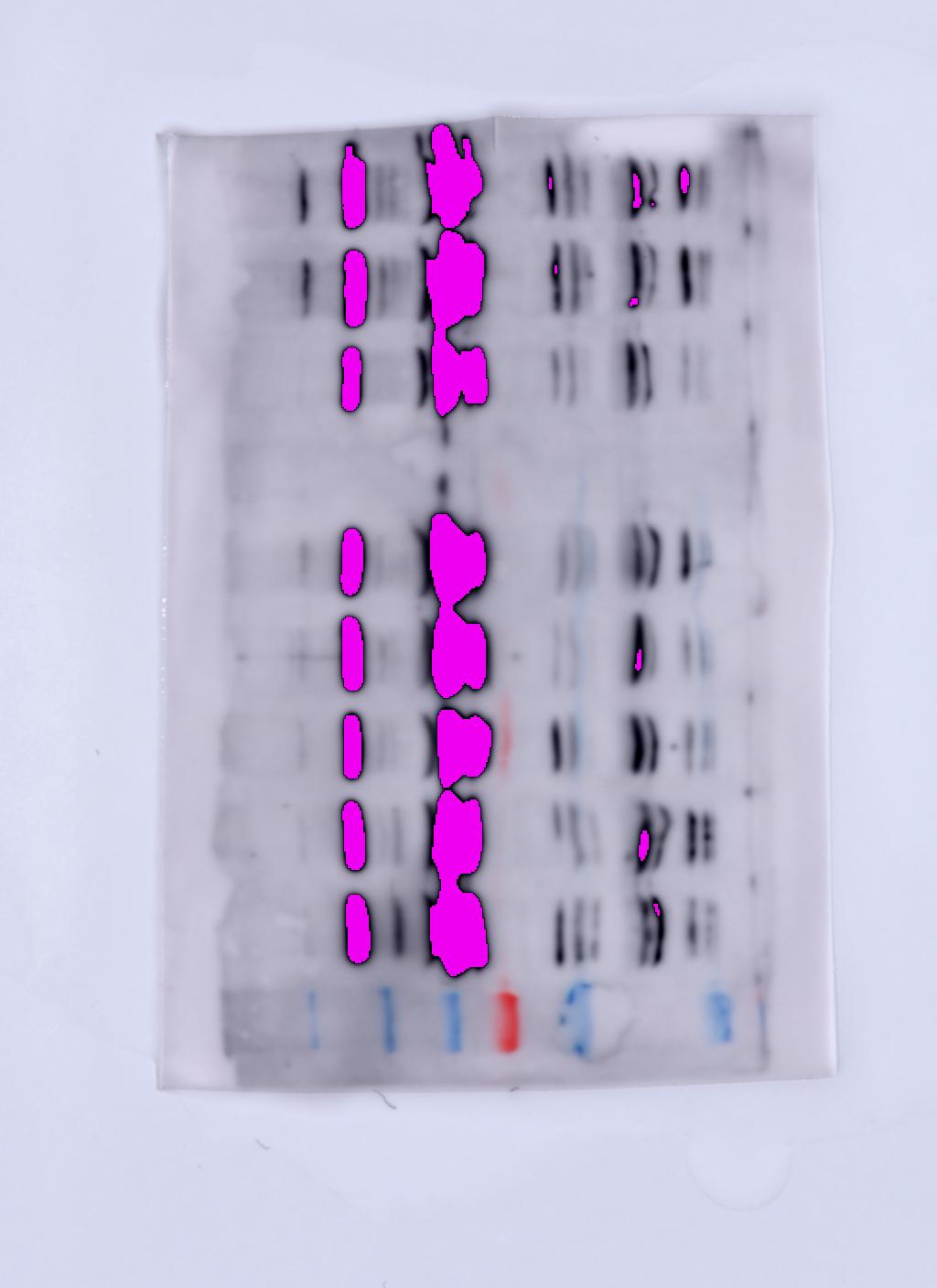

Supplement: Figure 2—source data 1. [file elife-87125-fig2-data1.zip › Figure 2-source data 1/Fig.2_sourceData 1_(1).jpg]

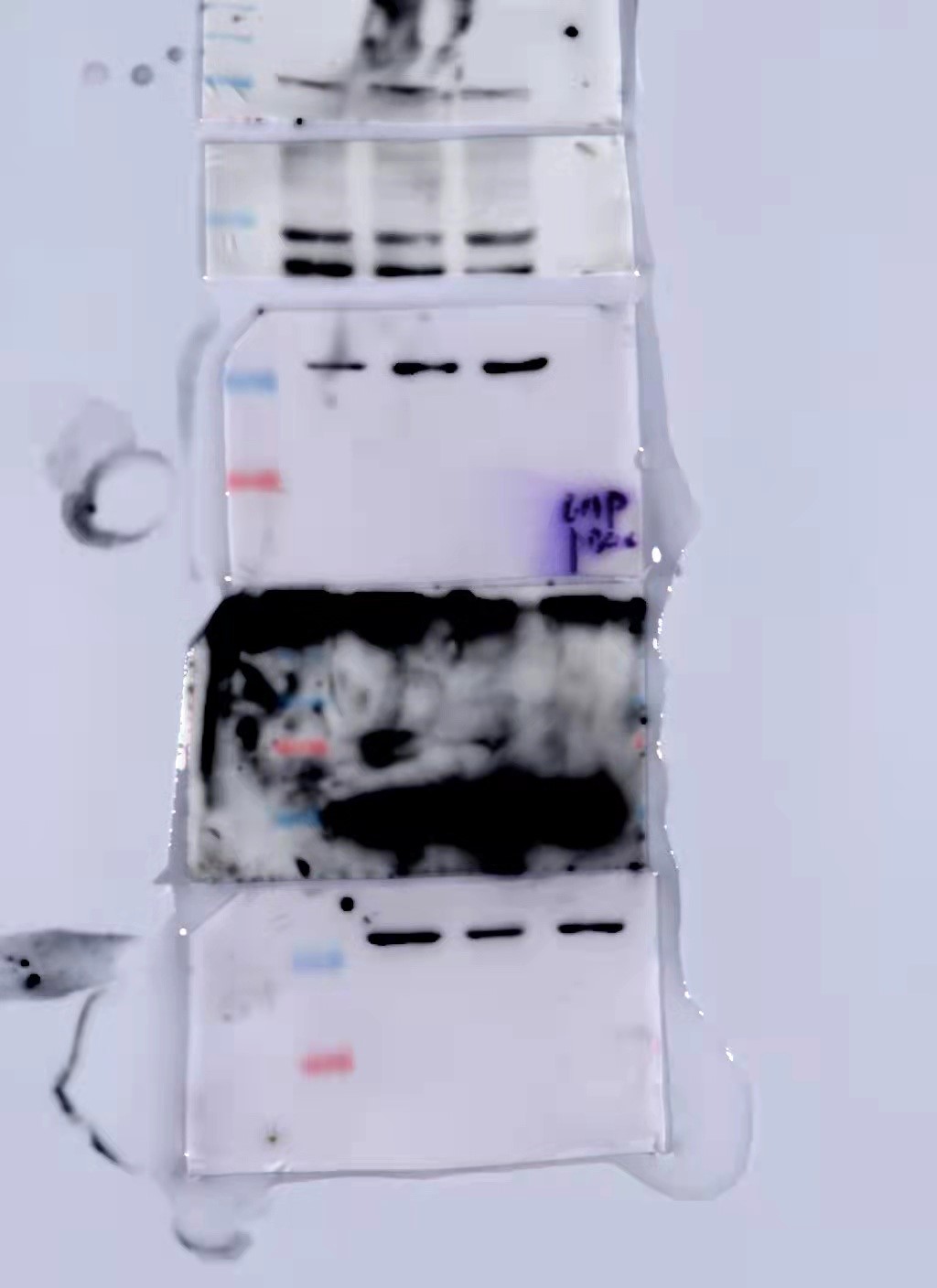

Supplement: Figure 3—source data 1. [file elife-87125-fig3-data1.zip › Figure 3-source data 1-4/Fig.3_Source data 1(9).jpg]

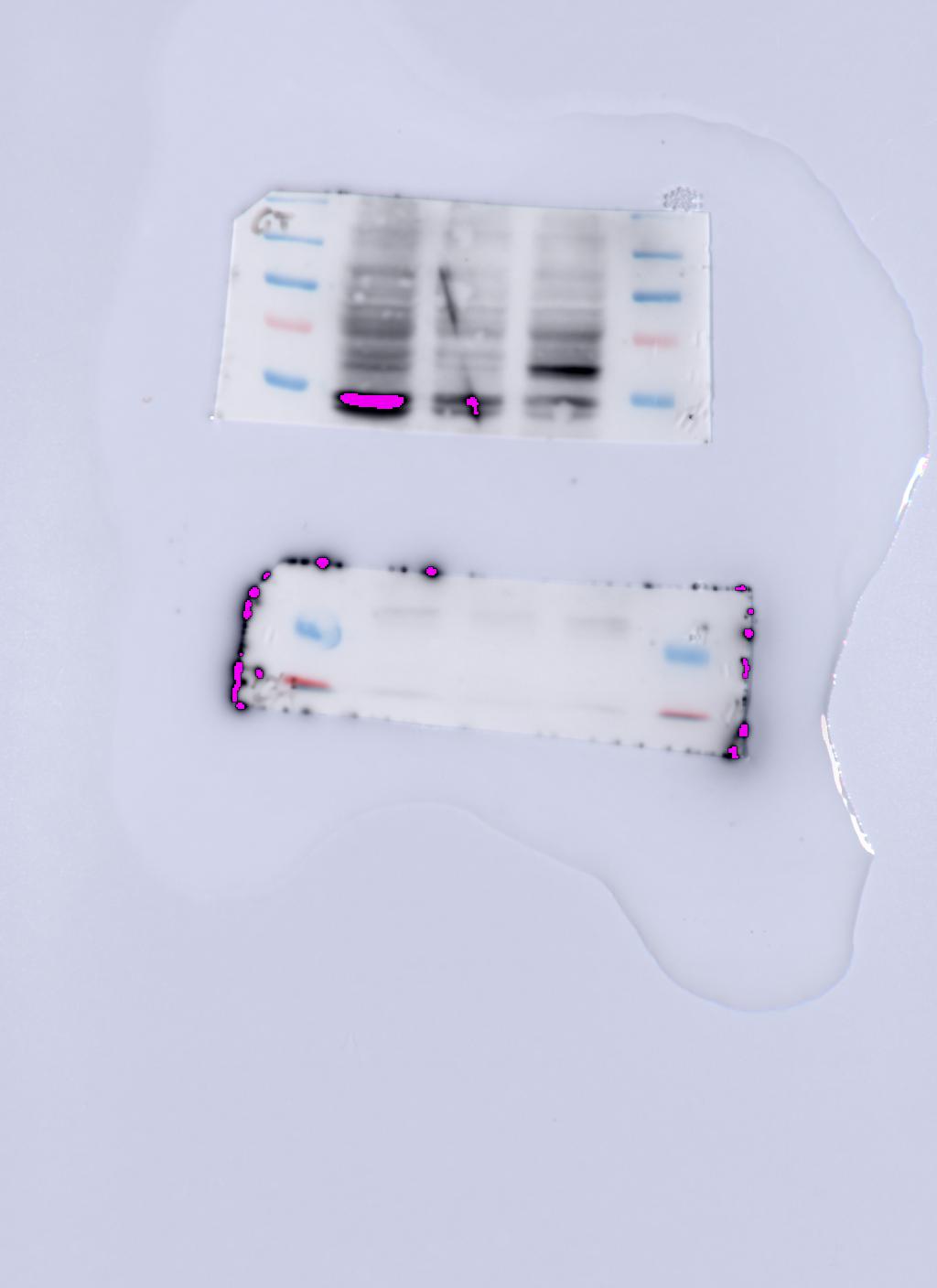

Supplement: Figure 3—source data 1. [file elife-87125-fig3-data1.zip › Figure 3-source data 1-4/Fig.3_Source data 1(5).jpg]

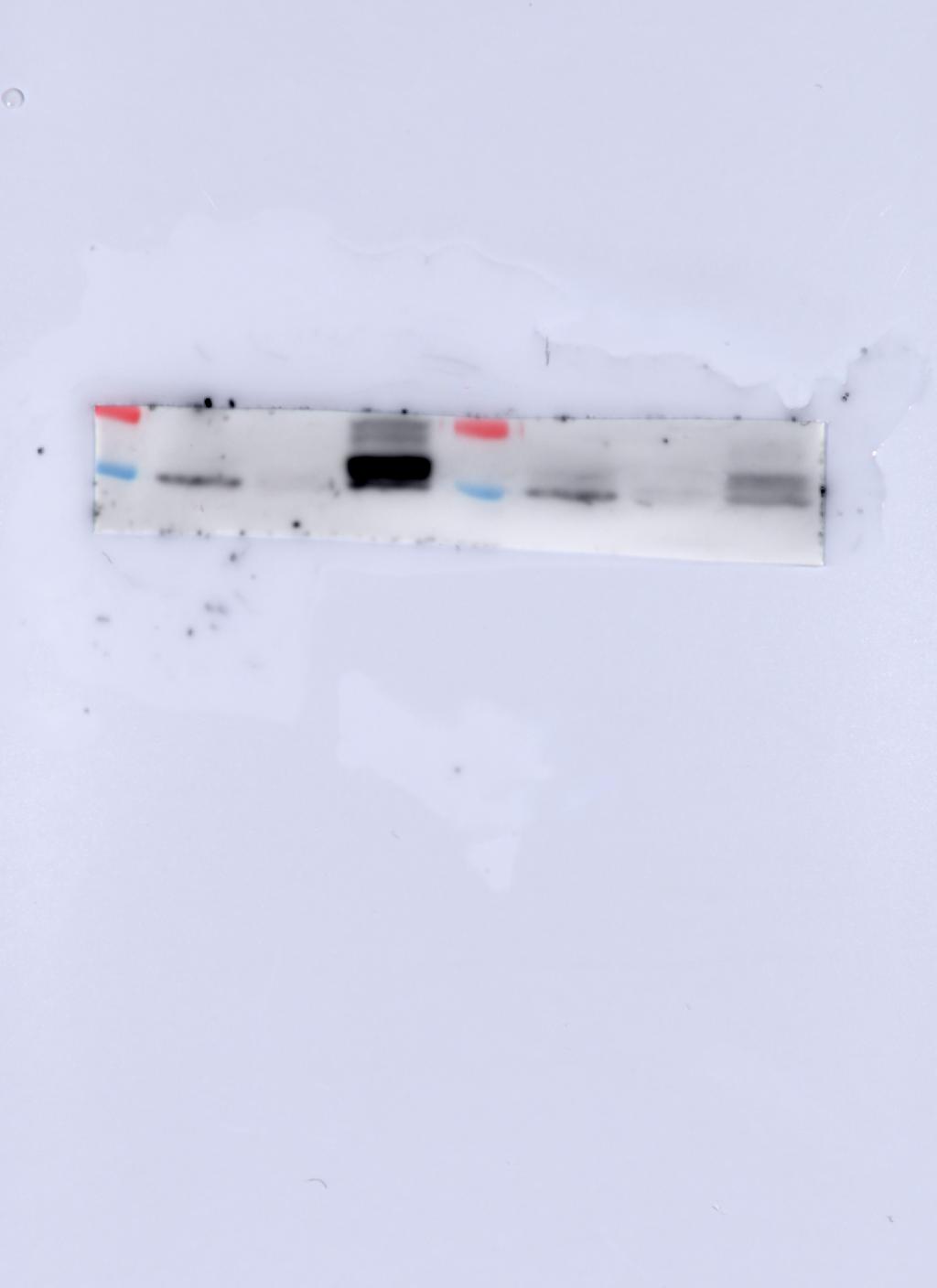

Supplement: Figure 3—source data 1. [file elife-87125-fig3-data1.zip › Figure 3-source data 1-4/Fig.3_Source data 2(1).jpg]

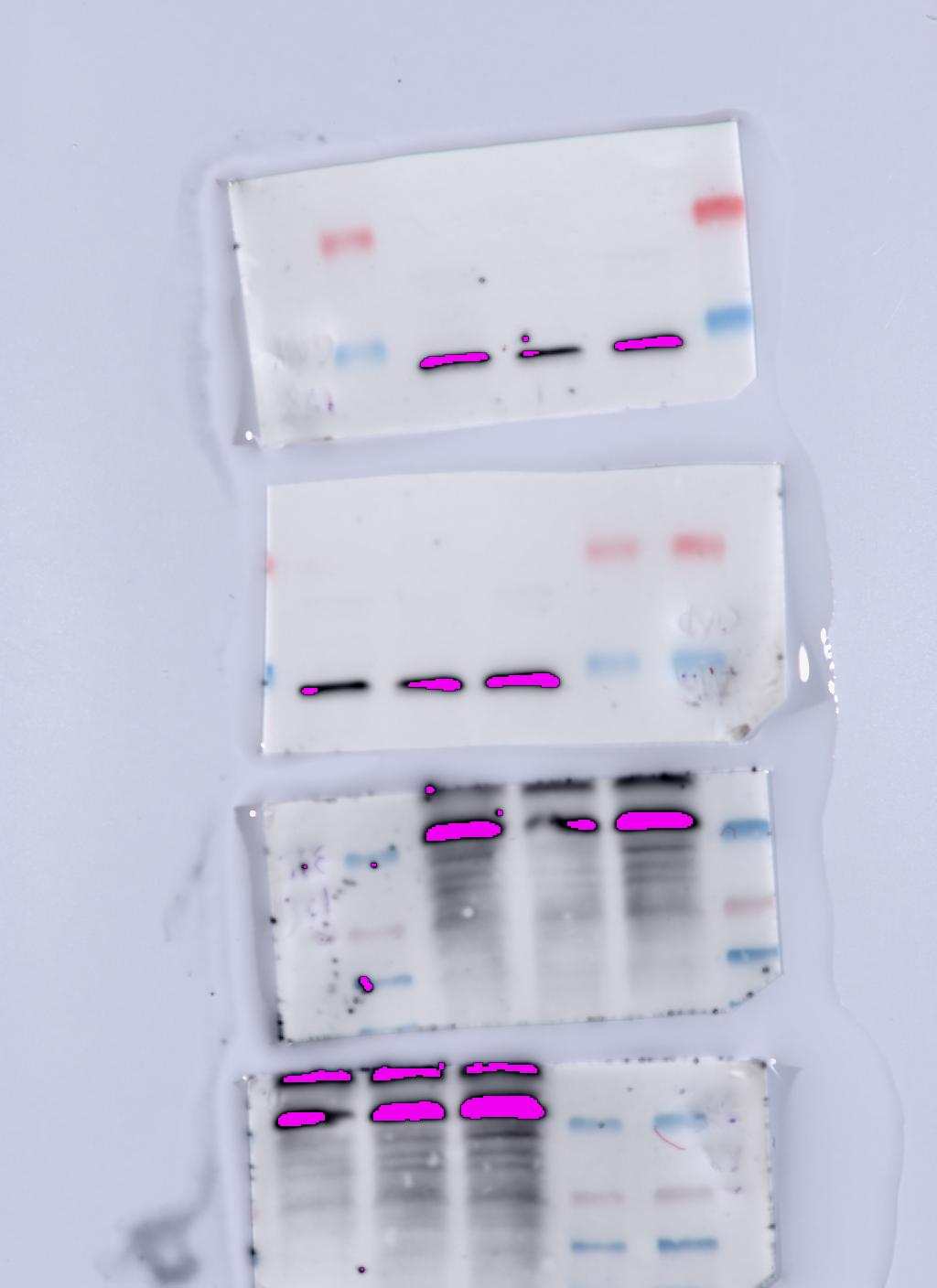

Supplement: Figure 3—source data 1. [file elife-87125-fig3-data1.zip › Figure 3-source data 1-4/Fig.3_Source data 1(4).jpg]

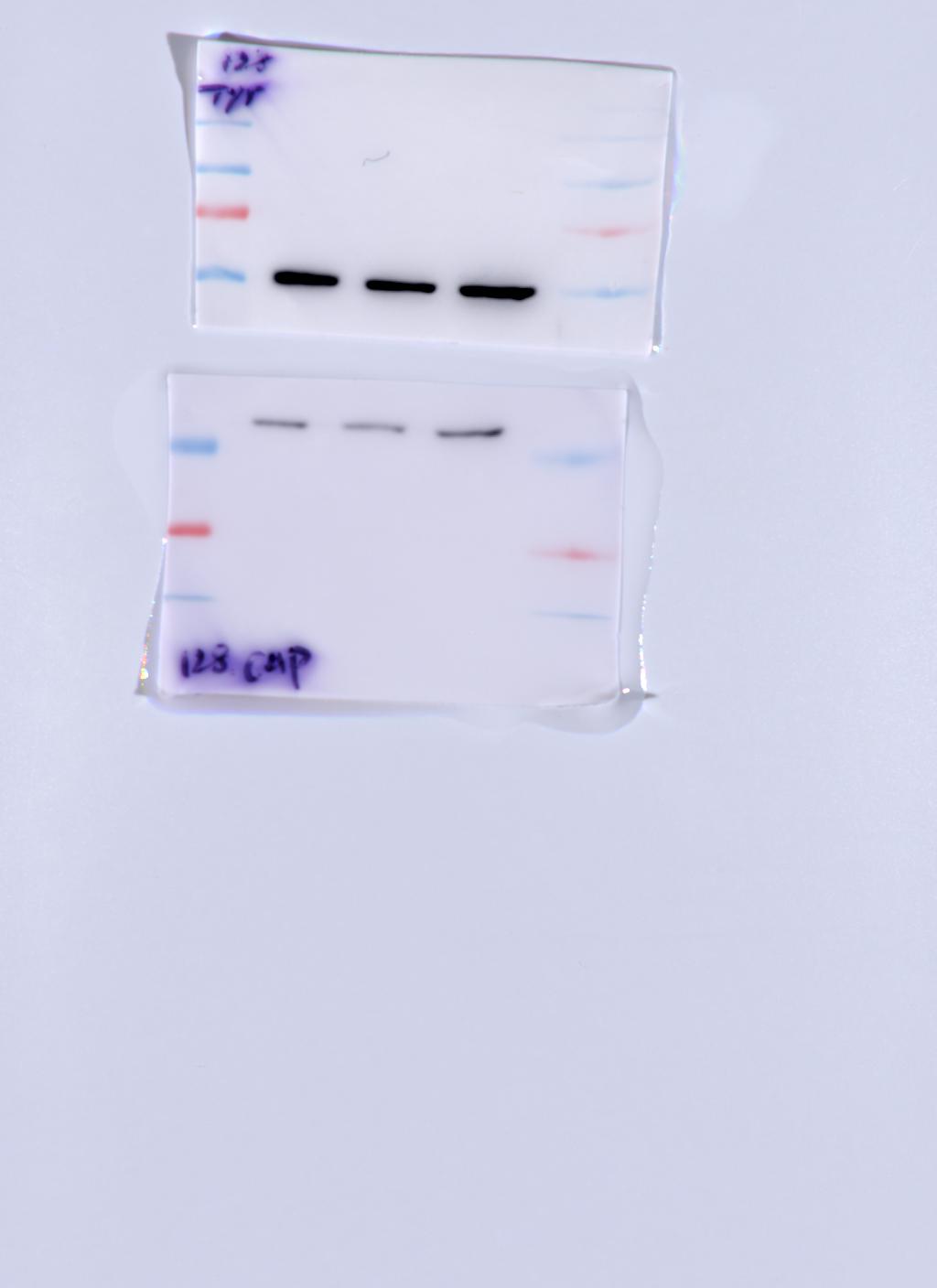

Supplement: Figure 3—source data 1. [file elife-87125-fig3-data1.zip › Figure 3-source data 1-4/Fig.3_Source data 1(8).jpg]

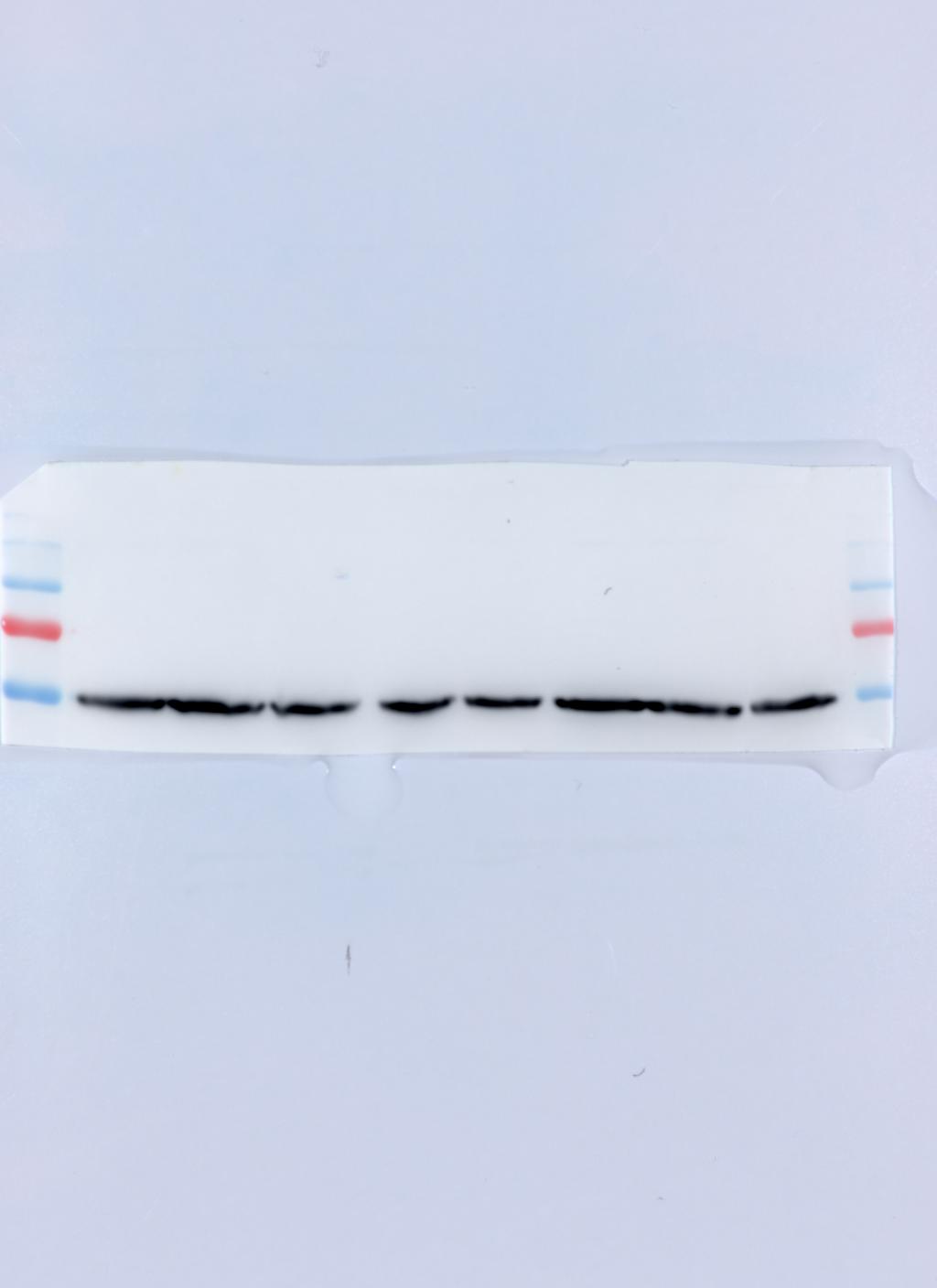

Supplement: Figure 3—source data 1. [file elife-87125-fig3-data1.zip › Figure 3-source data 1-4/Fig.3_Source data 3(2).jpg]

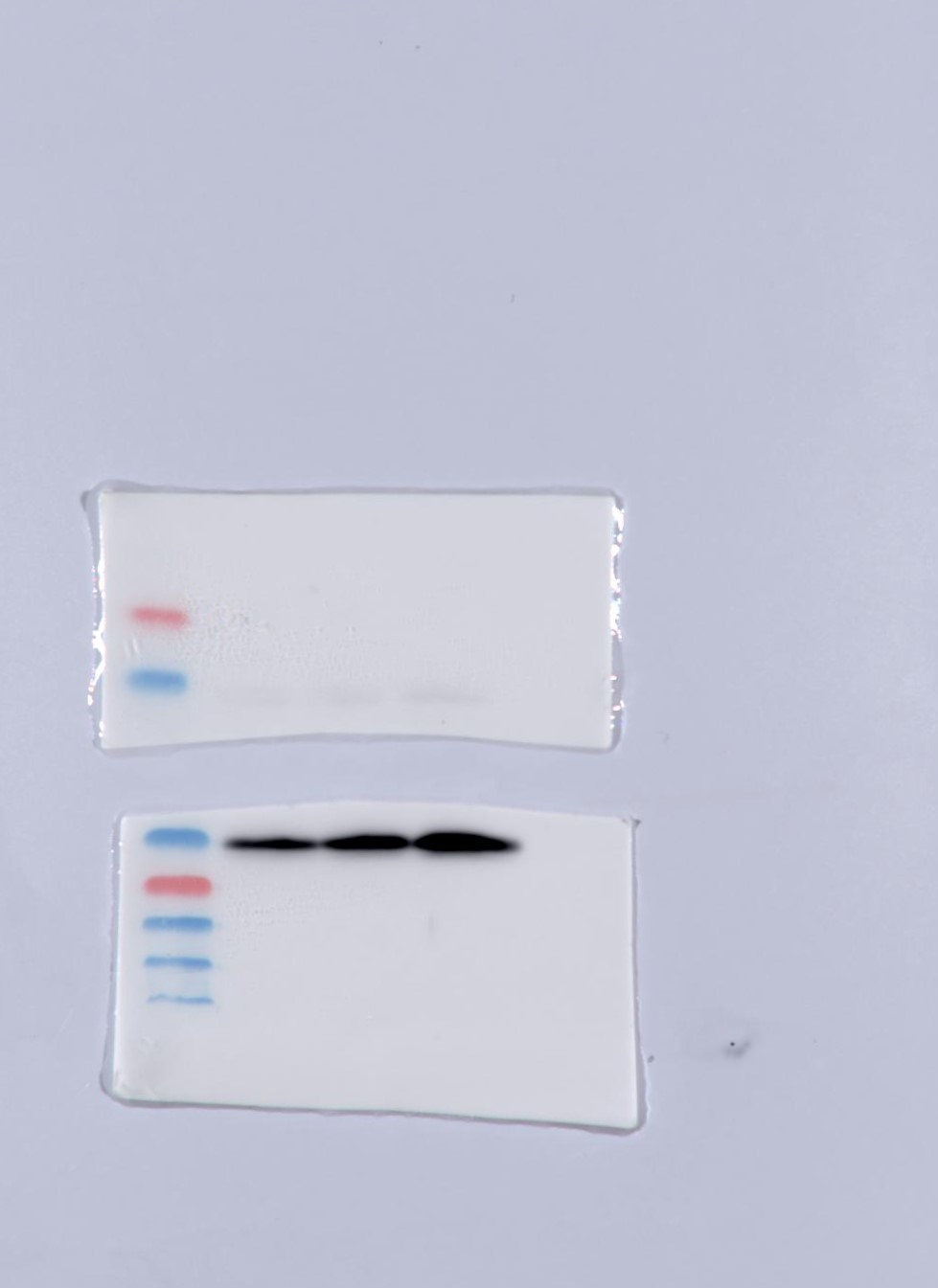

Supplement: Figure 3—source data 1. [file elife-87125-fig3-data1.zip › Figure 3-source data 1-4/Fig.3_Source data 2(6).jpg]

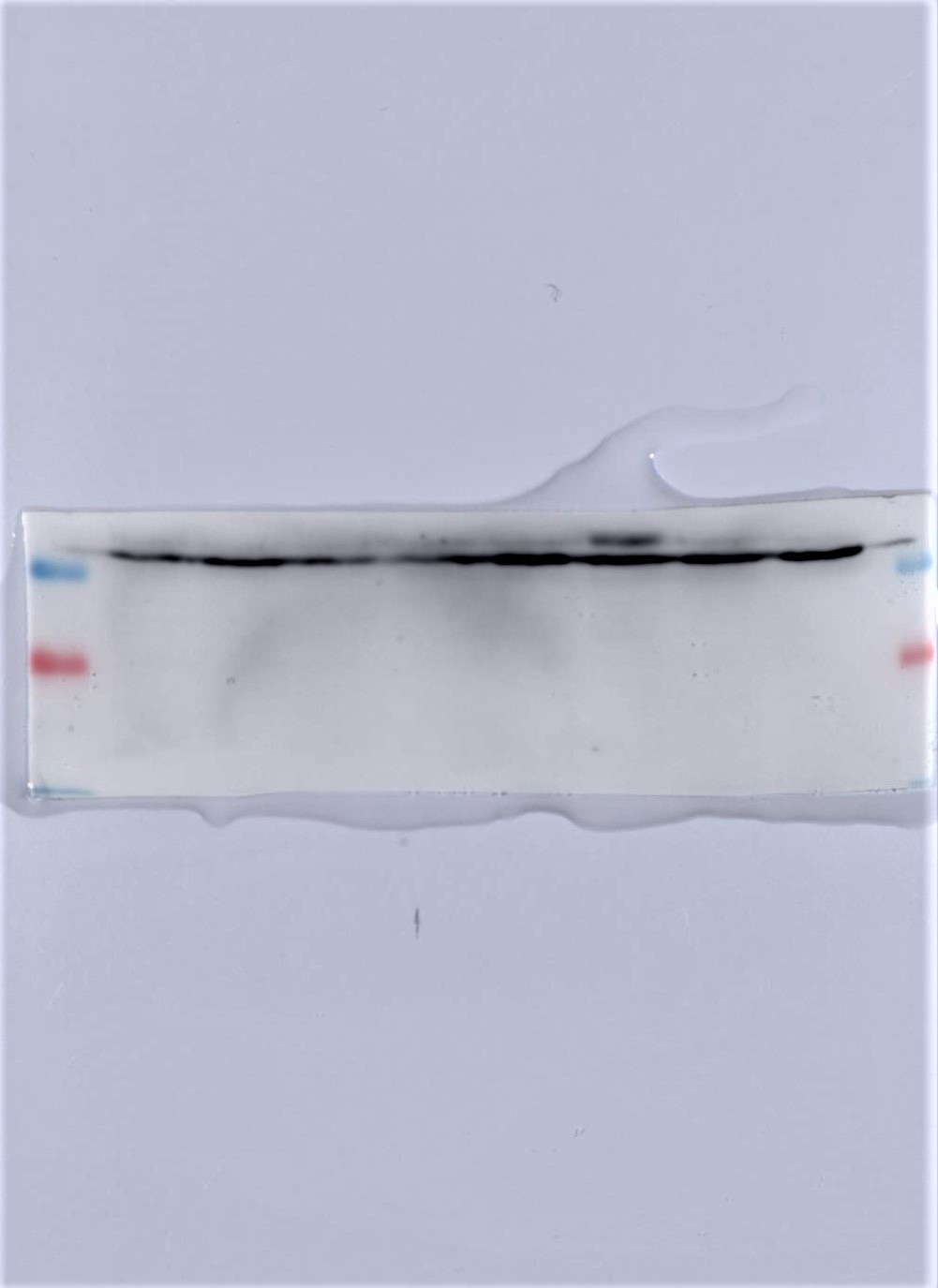

Supplement: Figure 3—source data 1. [file elife-87125-fig3-data1.zip › Figure 3-source data 1-4/Fig.3_Source data 3(3).jpg]

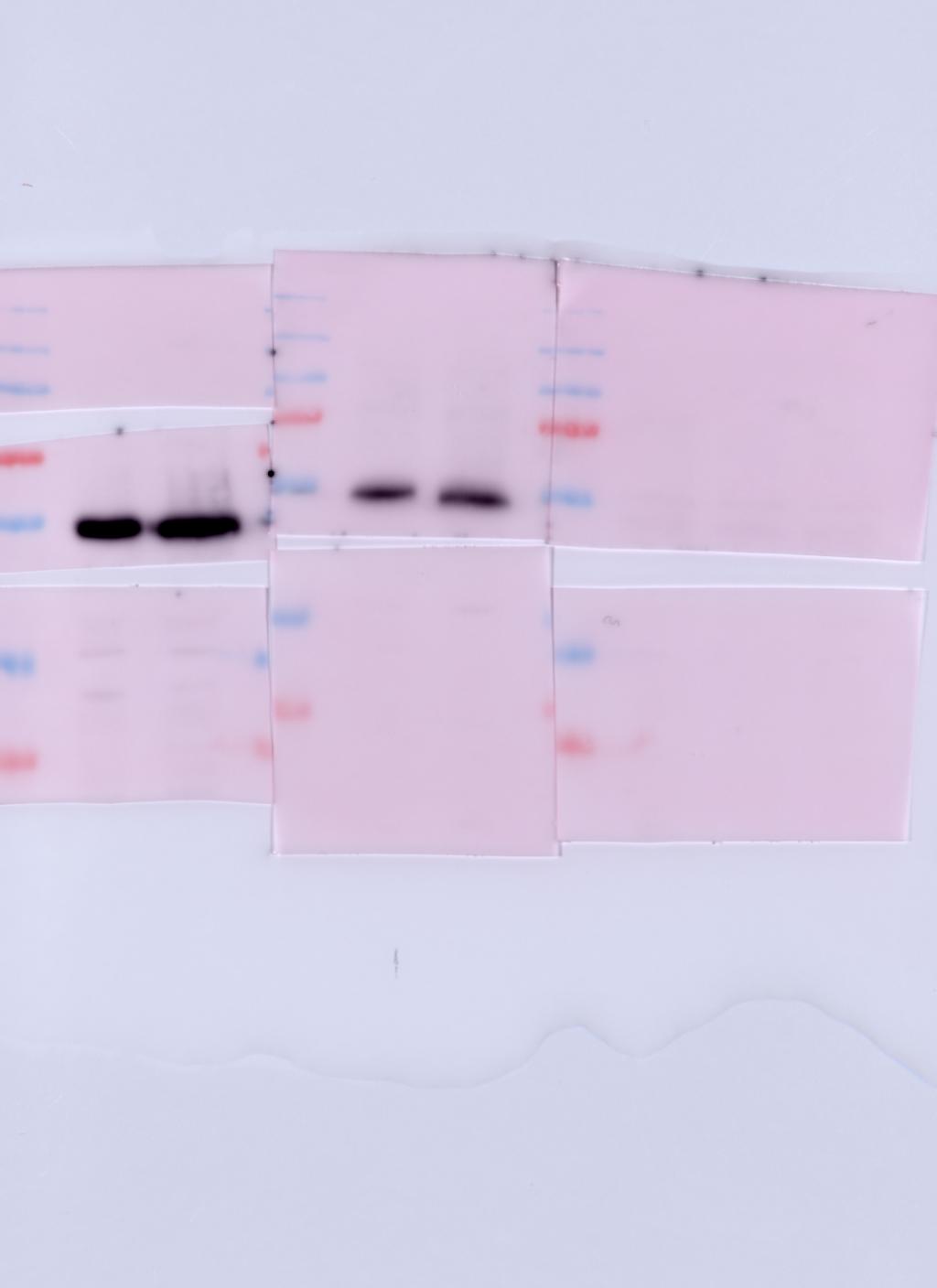

Supplement: Figure 3—source data 1. [file elife-87125-fig3-data1.zip › Figure 3-source data 1-4/Fig.3_Source data 4(1).jpg]

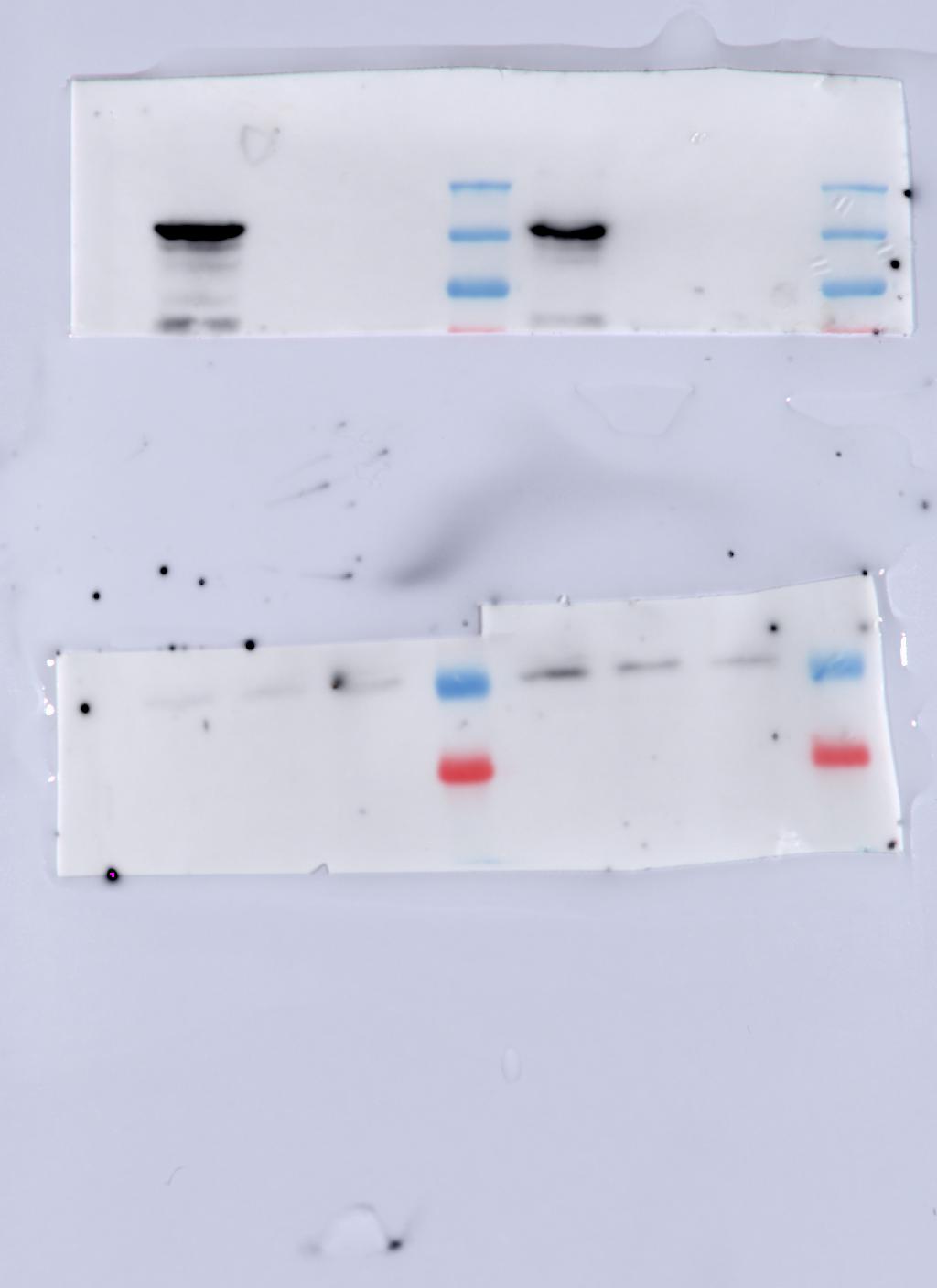

Supplement: Figure 3—source data 1. [file elife-87125-fig3-data1.zip › Figure 3-source data 1-4/Fig.3_Source data 1(2).jpg]

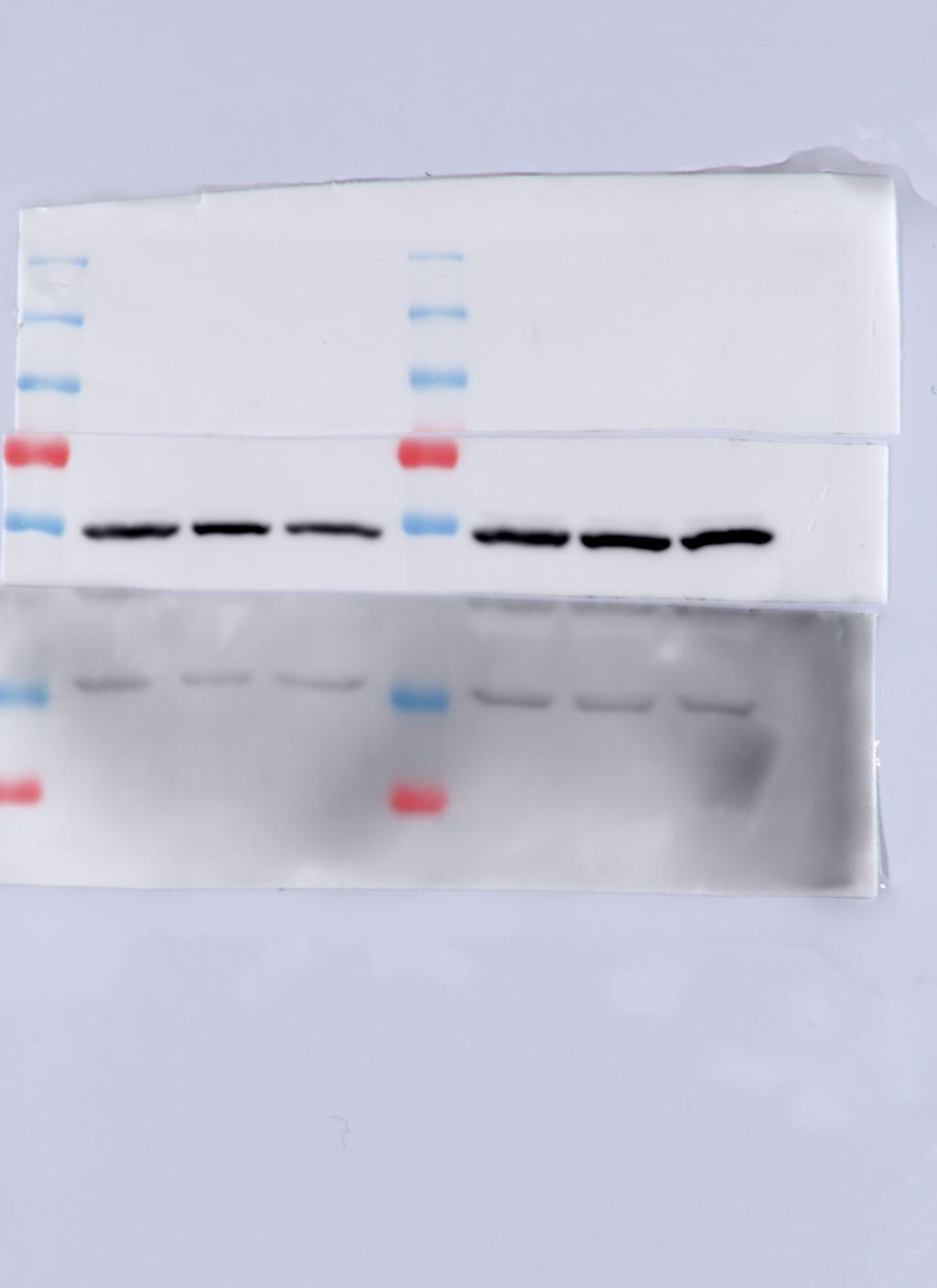

Supplement: Figure 3—source data 1. [file elife-87125-fig3-data1.zip › Figure 3-source data 1-4/Fig.3_Source data 2(4).jpg]

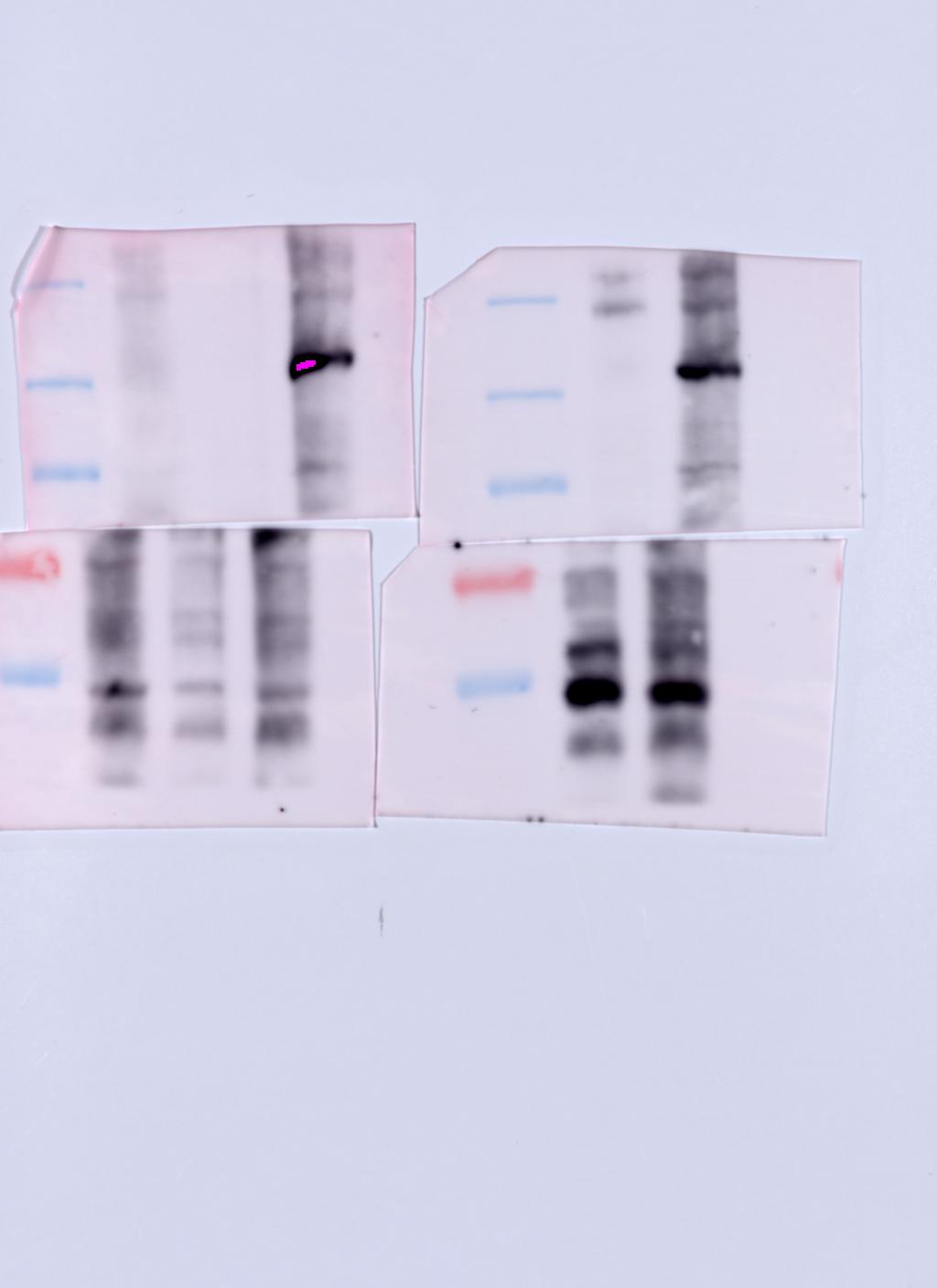

Supplement: Figure 3—source data 1. [file elife-87125-fig3-data1.zip › Figure 3-source data 1-4/Fig.3_Source data 1(3) and Fig.3_Source data 4(2).jpg]

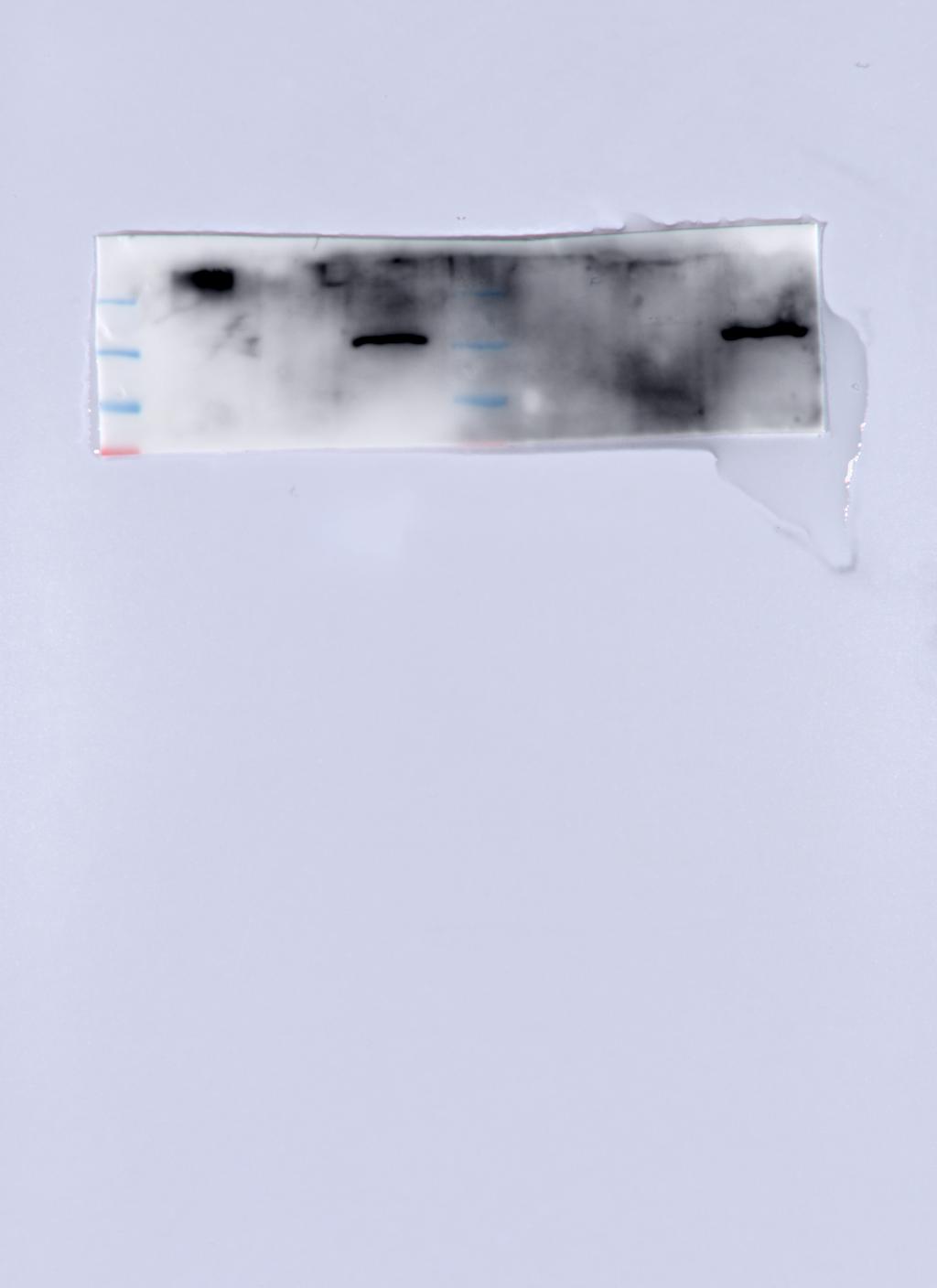

Supplement: Figure 3—source data 1. [file elife-87125-fig3-data1.zip › Figure 3-source data 1-4/Fig.3_Source data 1(1).jpg]

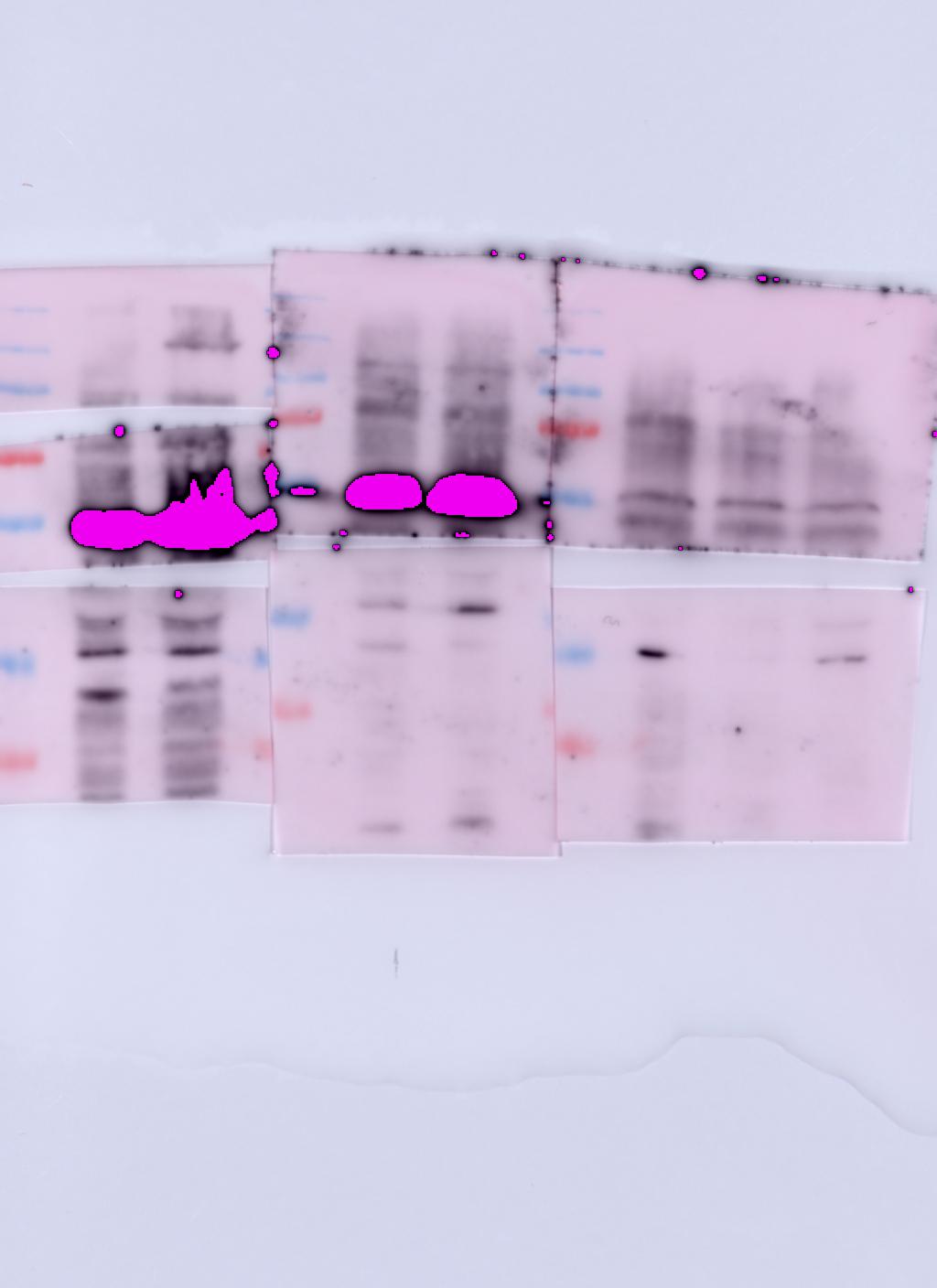

Supplement: Figure 3—source data 1. [file elife-87125-fig3-data1.zip › Figure 3-source data 1-4/Fig.3_Source data 4(3).jpg]

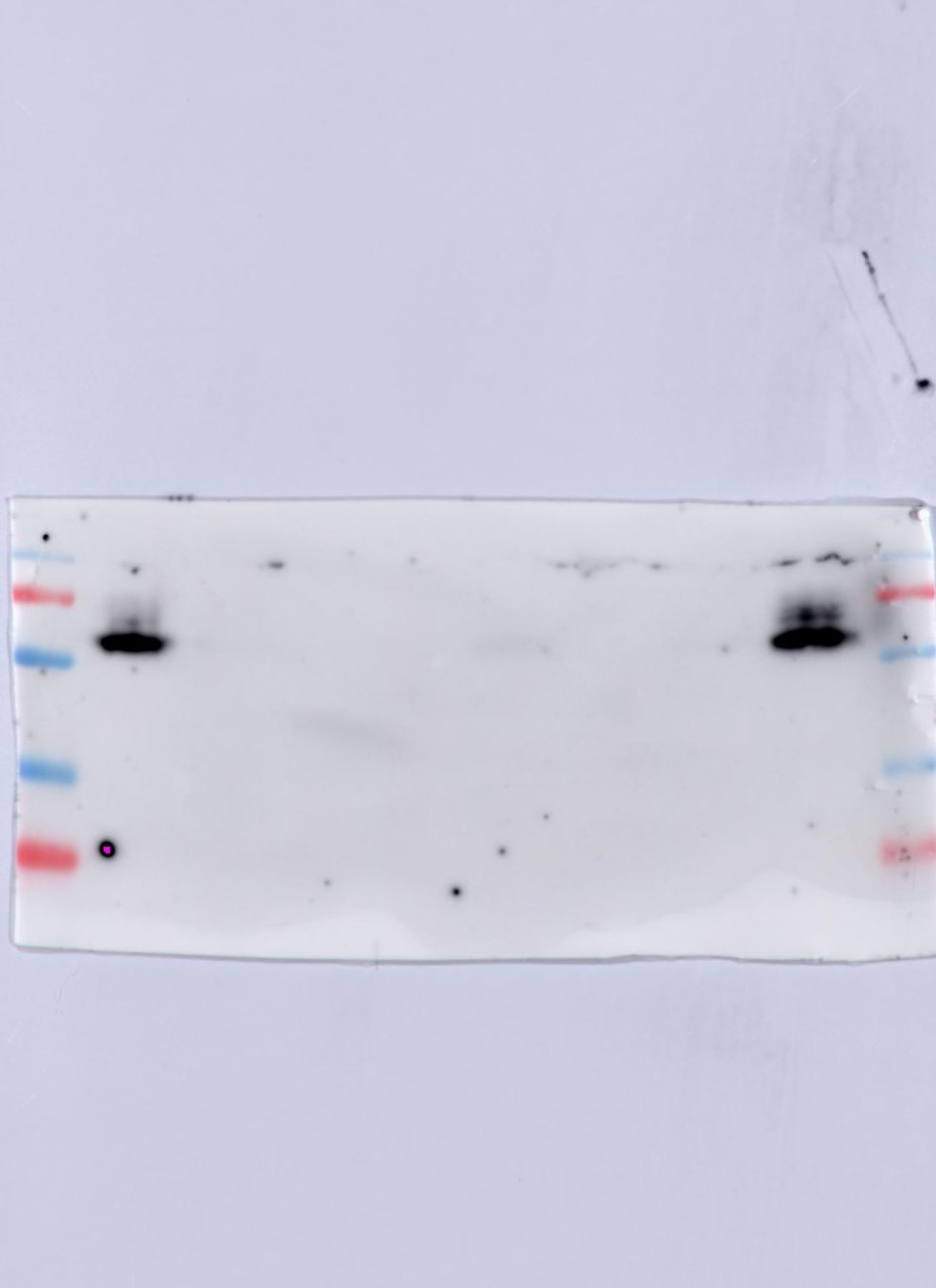

Supplement: Figure 3—source data 1. [file elife-87125-fig3-data1.zip › Figure 3-source data 1-4/Fig.3_Source data 3(1).jpg]

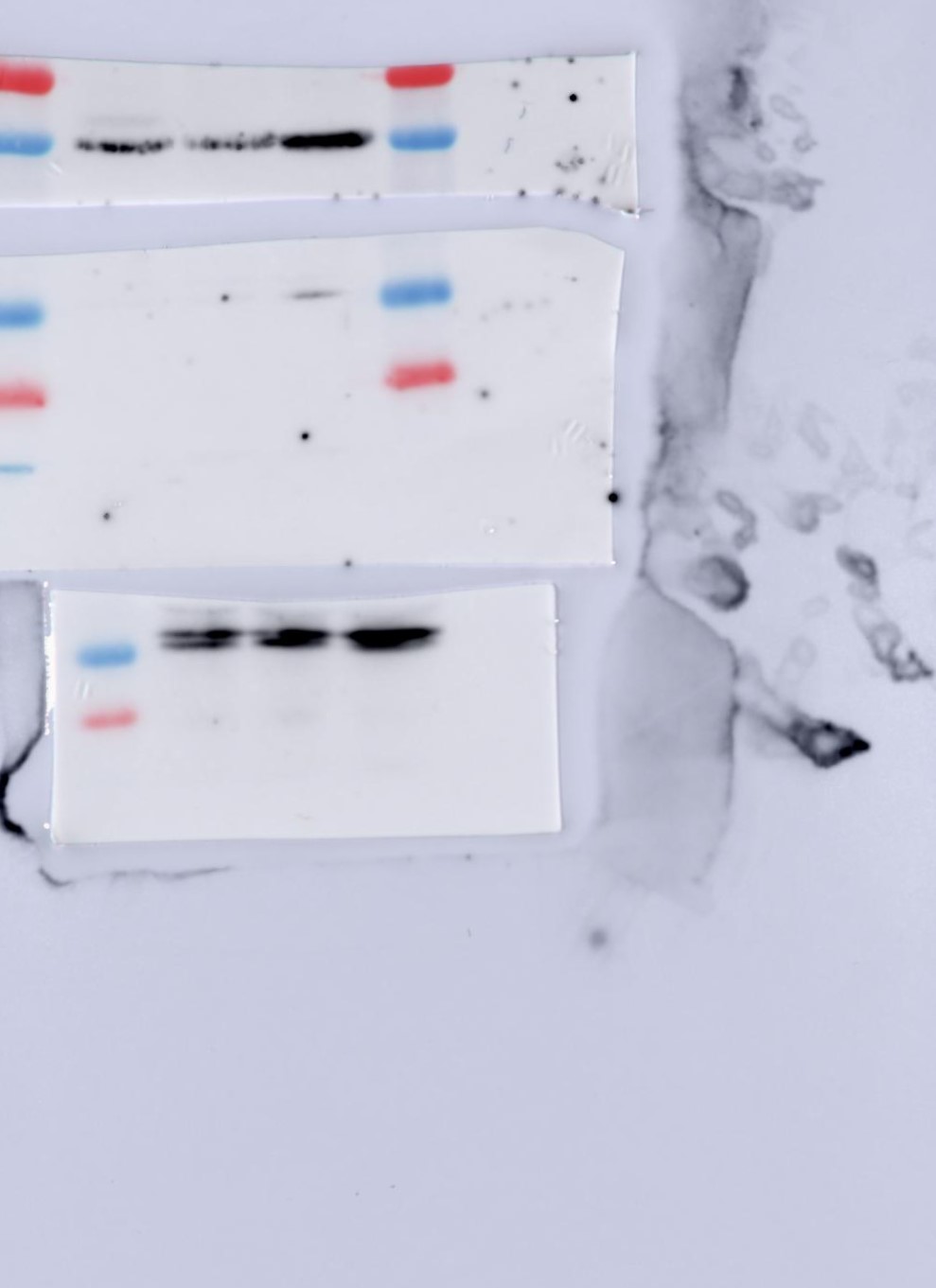

Supplement: Figure 3—source data 1. [file elife-87125-fig3-data1.zip › Figure 3-source data 1-4/Fig.3_Source data 2(5).jpg]

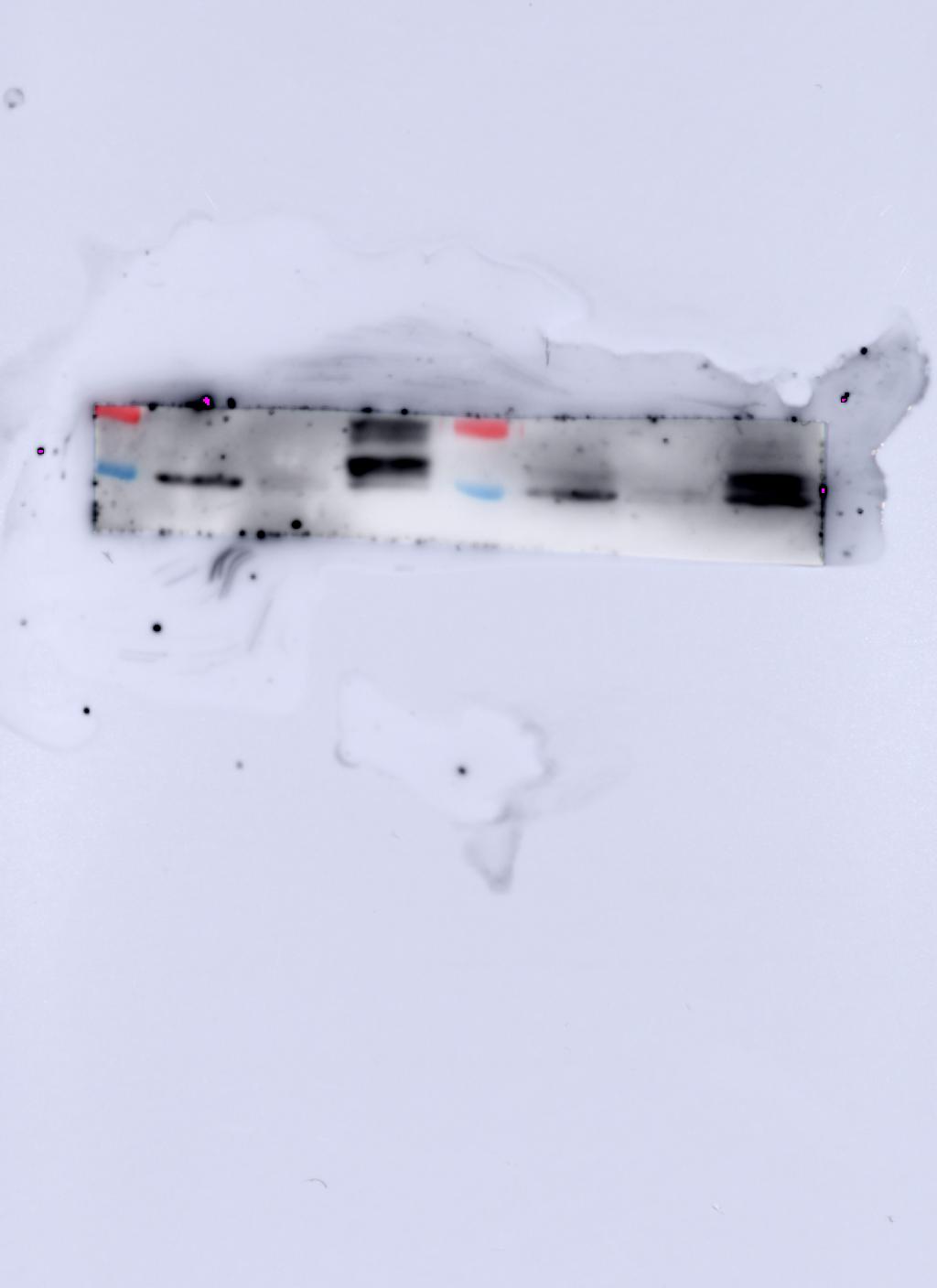

Supplement: Figure 3—source data 1. [file elife-87125-fig3-data1.zip › Figure 3-source data 1-4/Fig.3_Source data 2(2).jpg]

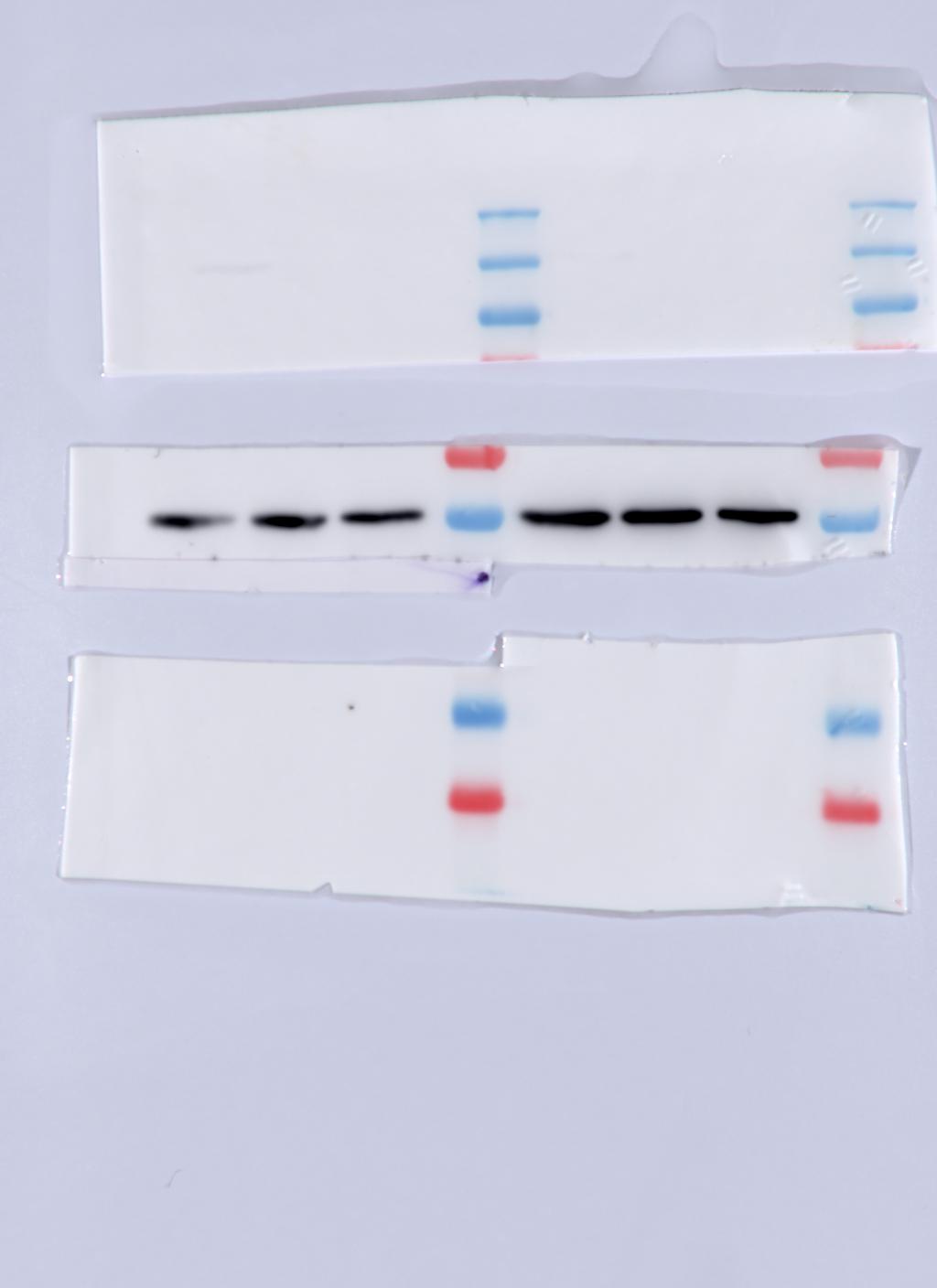

Supplement: Figure 3—source data 1. [file elife-87125-fig3-data1.zip › Figure 3-source data 1-4/Fig.3_Source data 1(7).jpg]

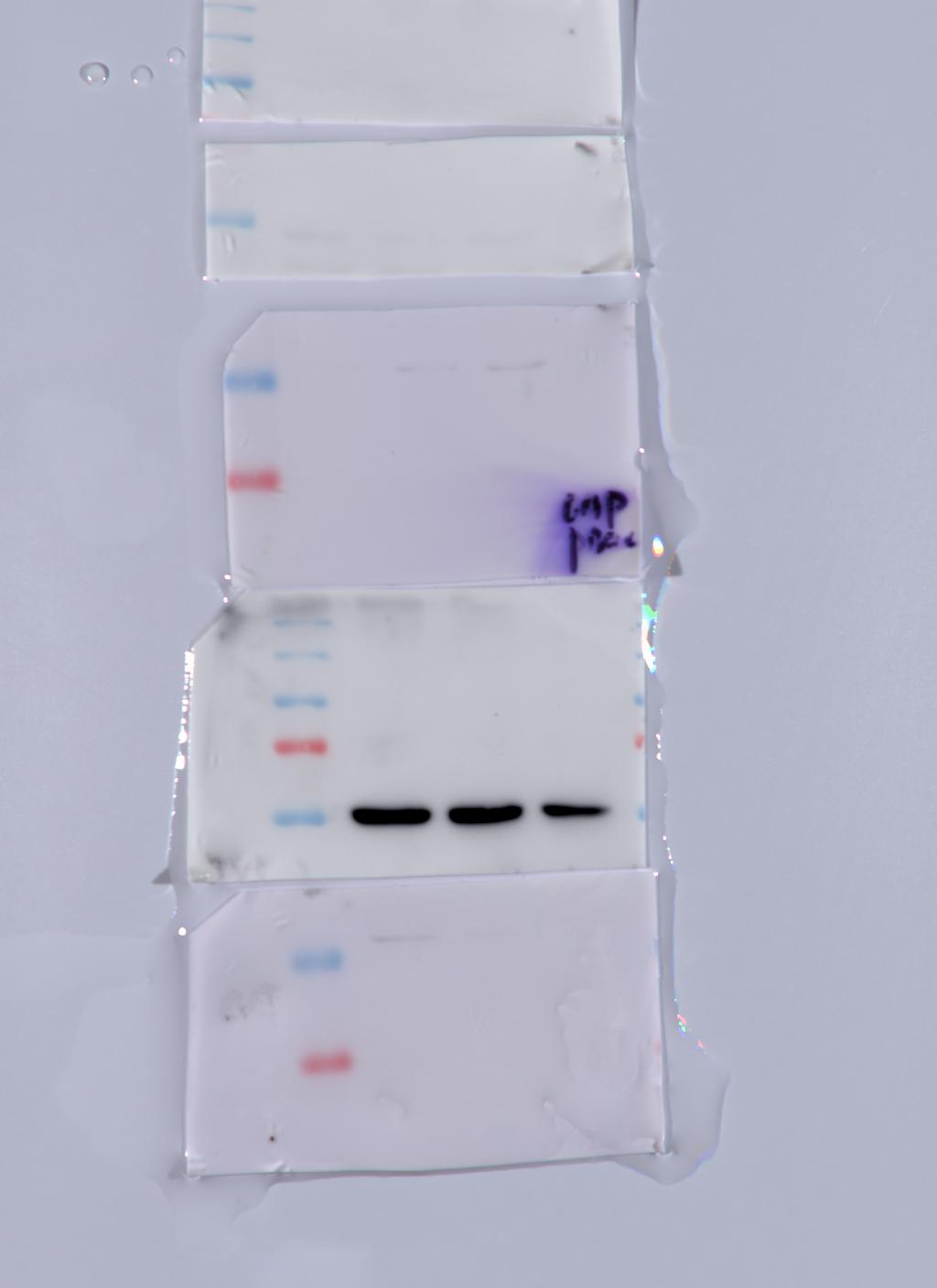

Supplement: Figure 3—source data 1. [file elife-87125-fig3-data1.zip › Figure 3-source data 1-4/Fig.3_Source data 1(6).jpg]

SourceDataFIG. 3B

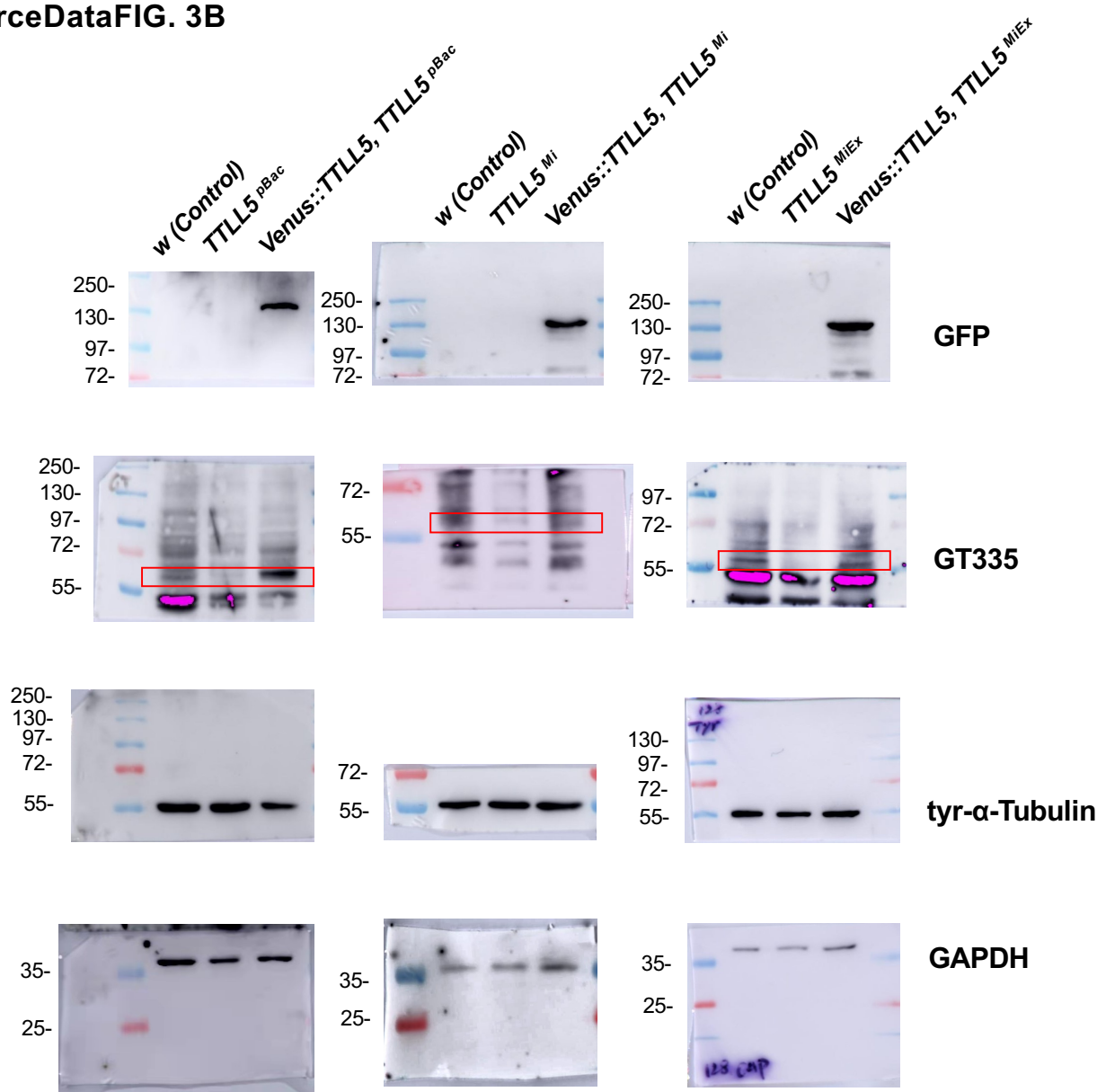

SourceDataFIG. 3D

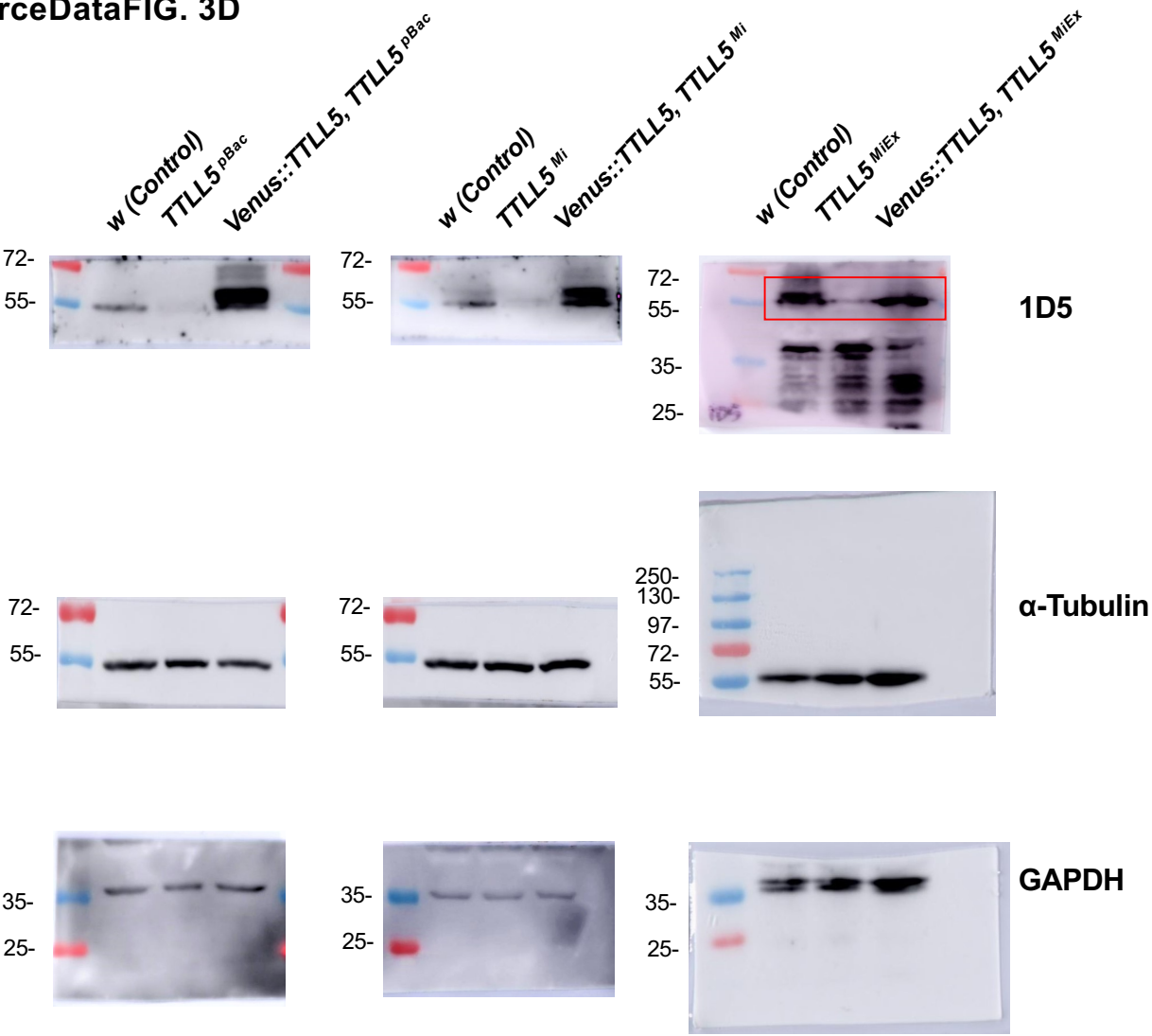

## SourceDataFIG. 3F

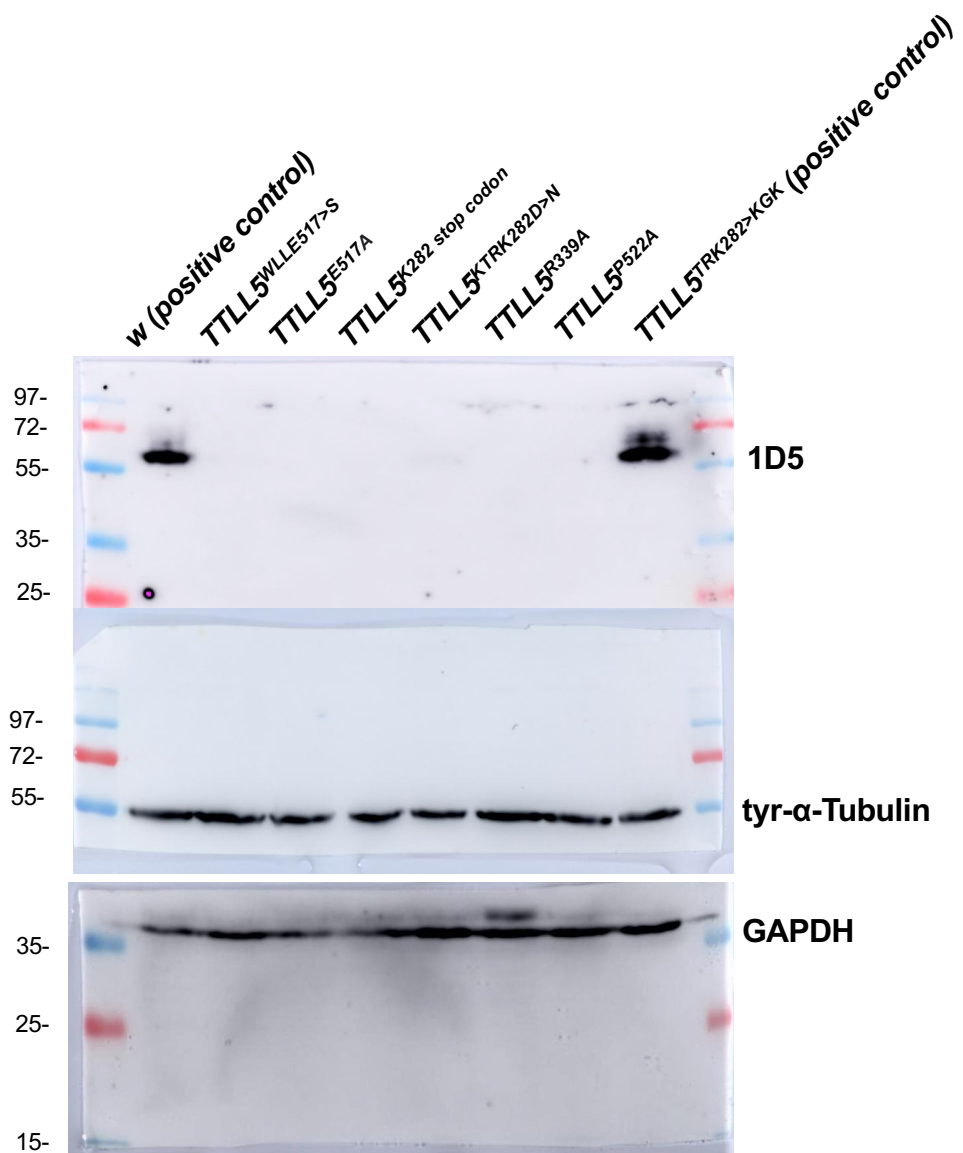

SourceDataFIG. 3G

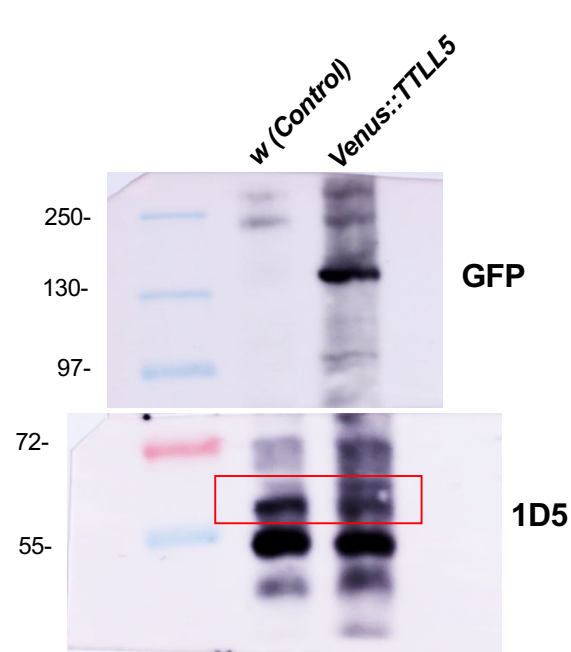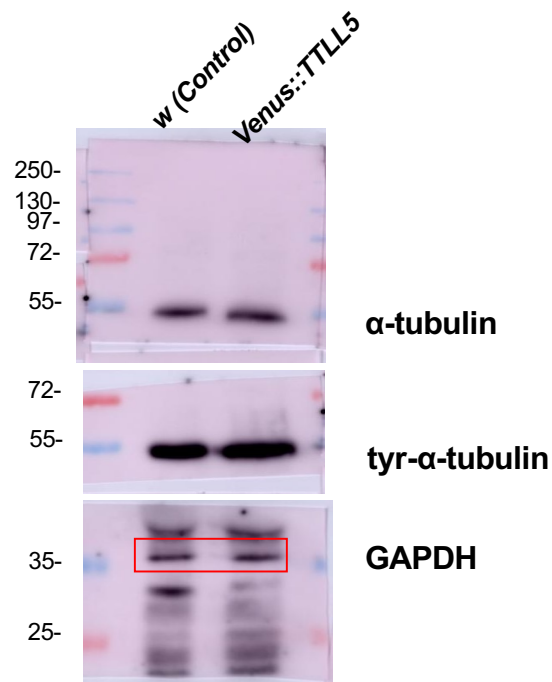

Supplement: Figure 3—source data 1. [file elife-87125-fig3-data1.zip › Figure 3-source data 1-4/Fig. 3_SourceData 1-4.pdf]

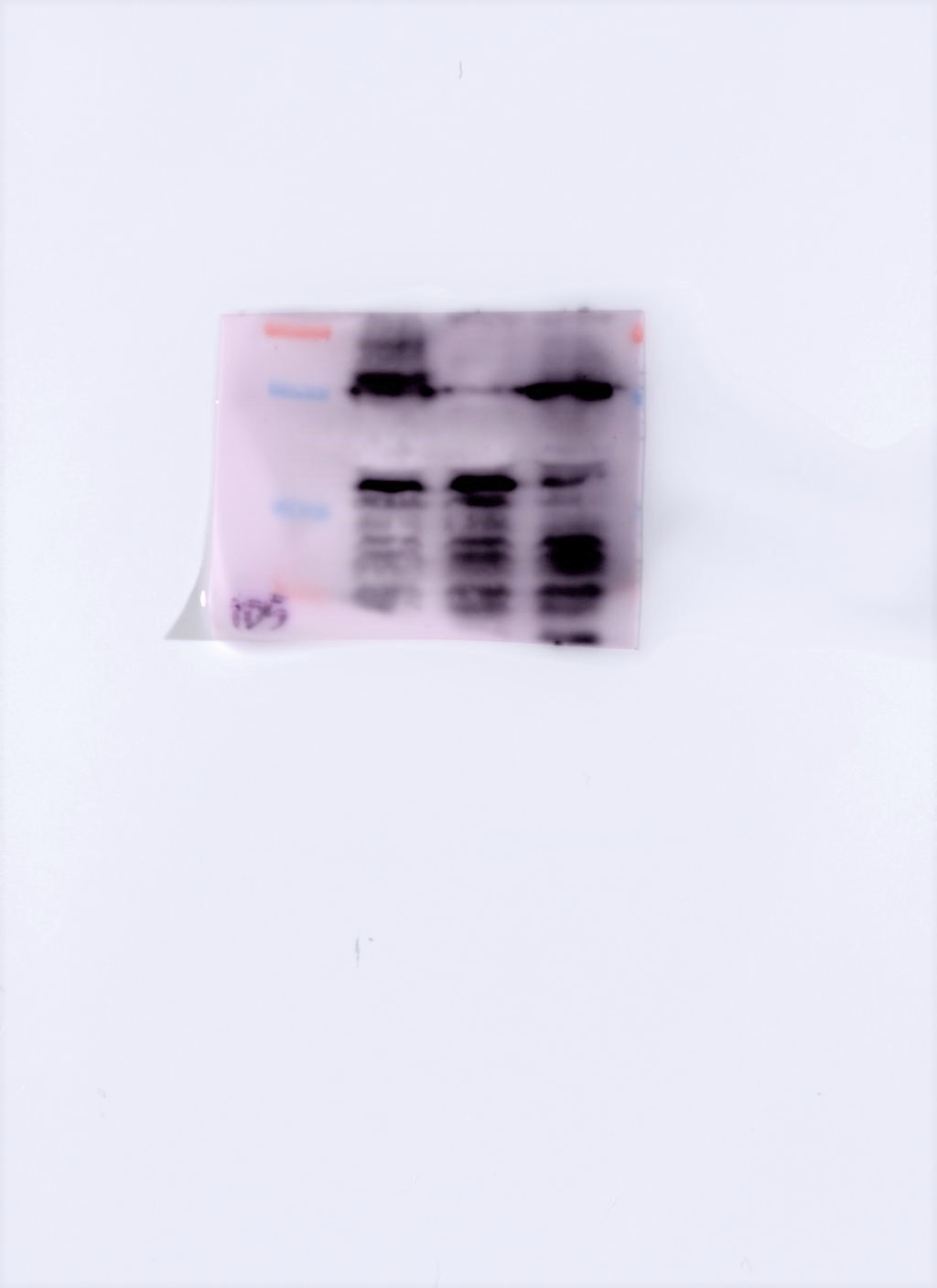

Supplement: Figure 3—source data 1. [file elife-87125-fig3-data1.zip › Figure 3-source data 1-4/Fig.3_Source data 2(3).jpg]

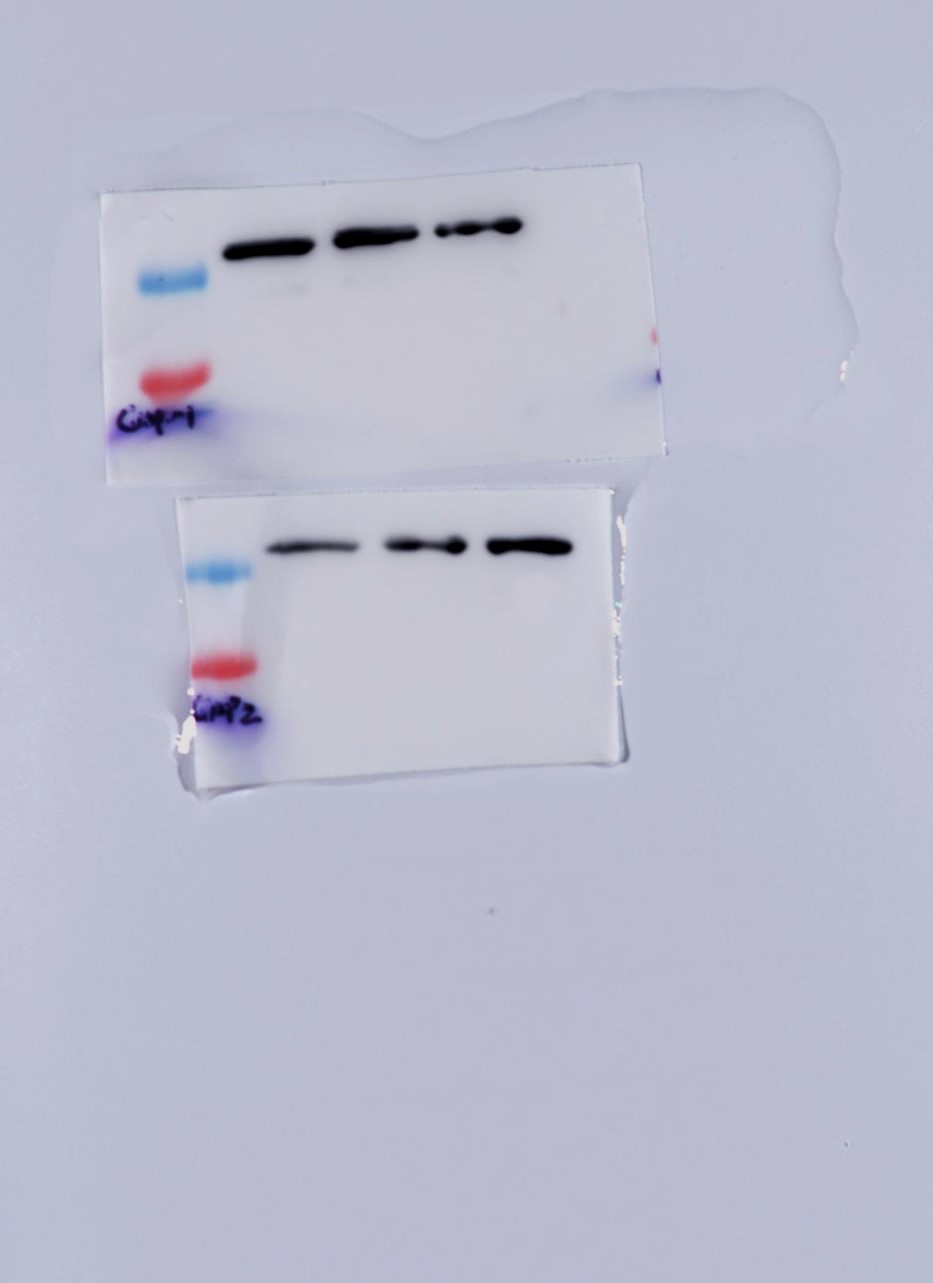

Supplement: Figure 7—source data 1. [file elife-87125-fig7-data1.zip › Figure 7-SourceData 1-2/Fig.7_Source data 1(3).jpg]

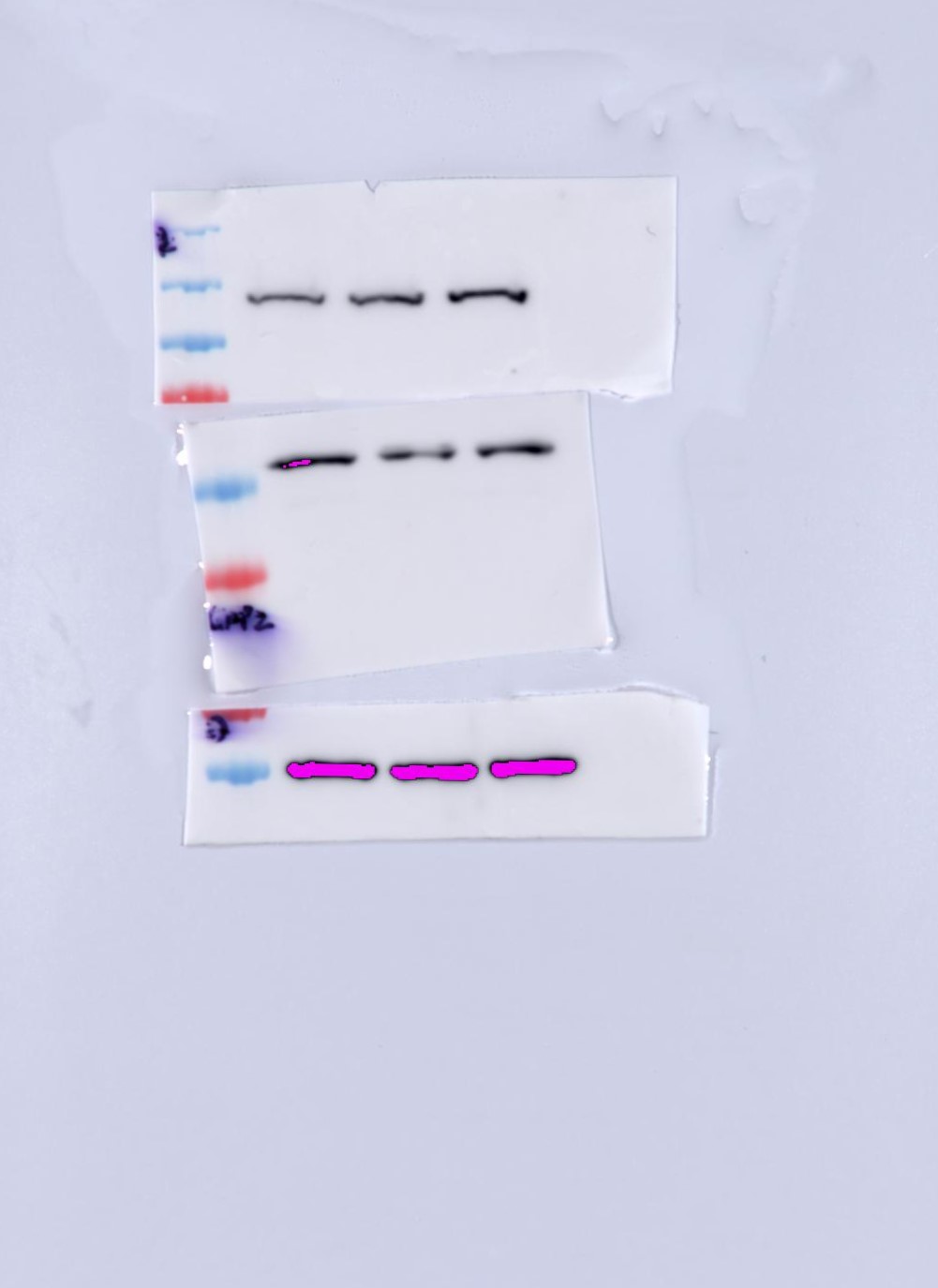

Supplement: Figure 7—source data 1. [file elife-87125-fig7-data1.zip › Figure 7-SourceData 1-2/Fig.7_Source data 1(2).jpg]

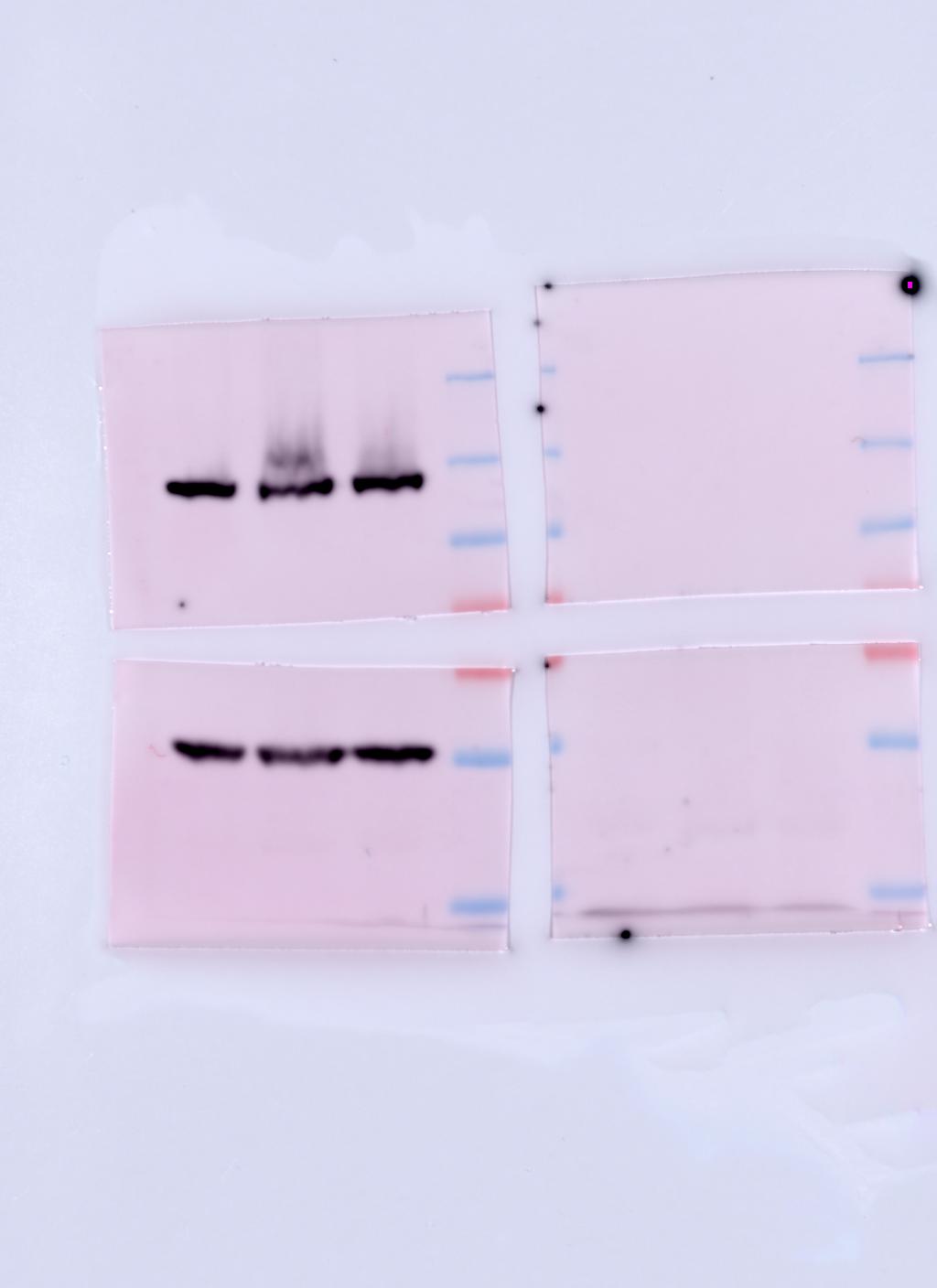

Supplement: Figure 7—source data 1. [file elife-87125-fig7-data1.zip › Figure 7-SourceData 1-2/Fig.7_Source data 1(5).jpg]

SourceDataFIG. 7A

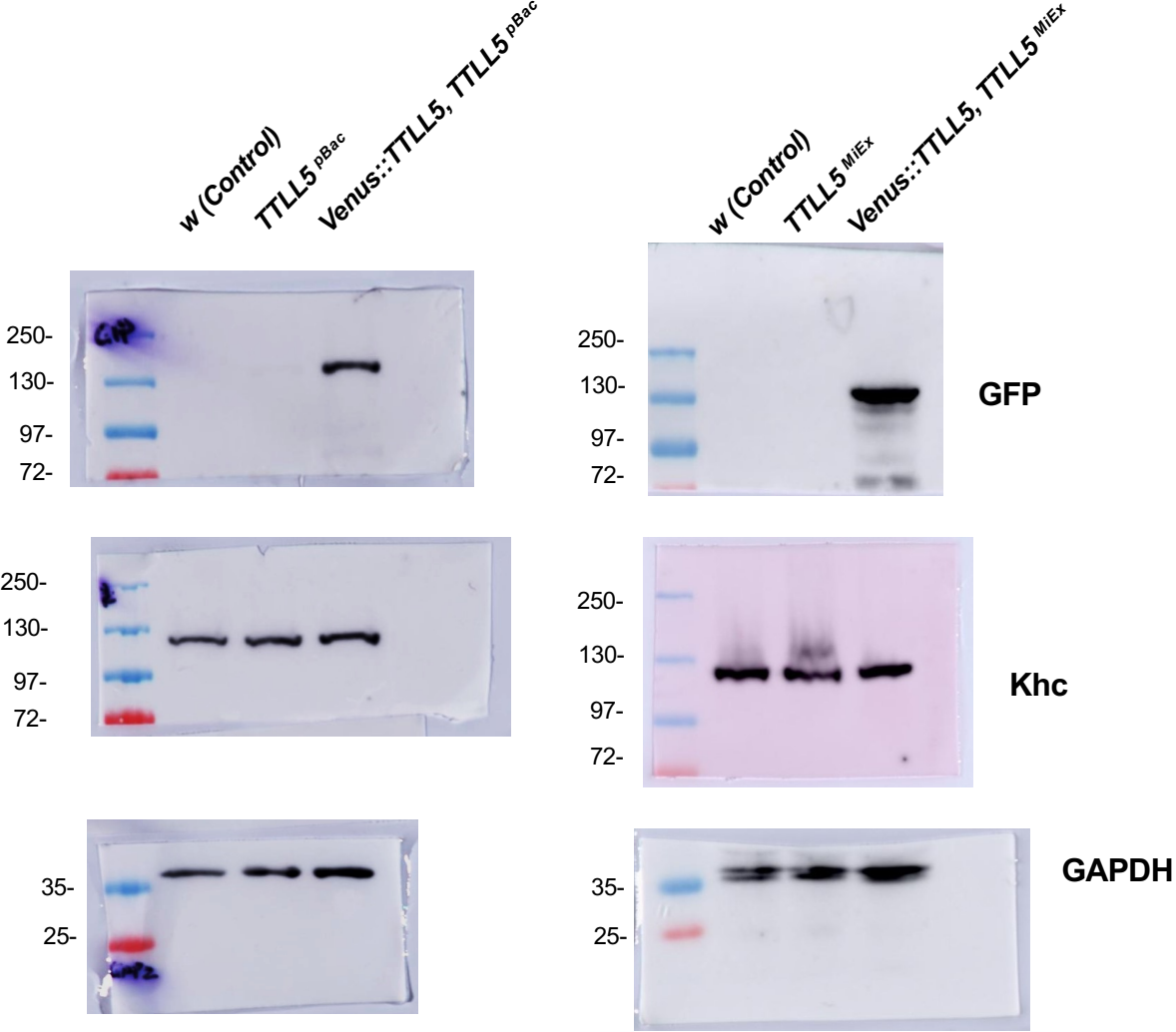

SourceDataFIG. 7B

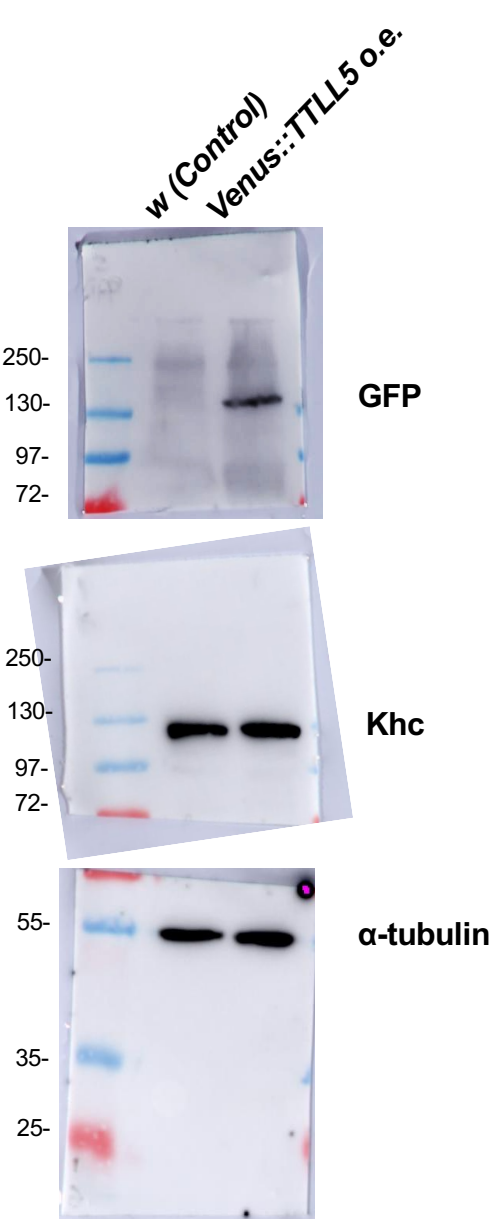

Supplement: Figure 7—source data 1. [file elife-87125-fig7-data1.zip › Figure 7-SourceData 1-2/Fig. 7_SourceData 1-2.pdf]

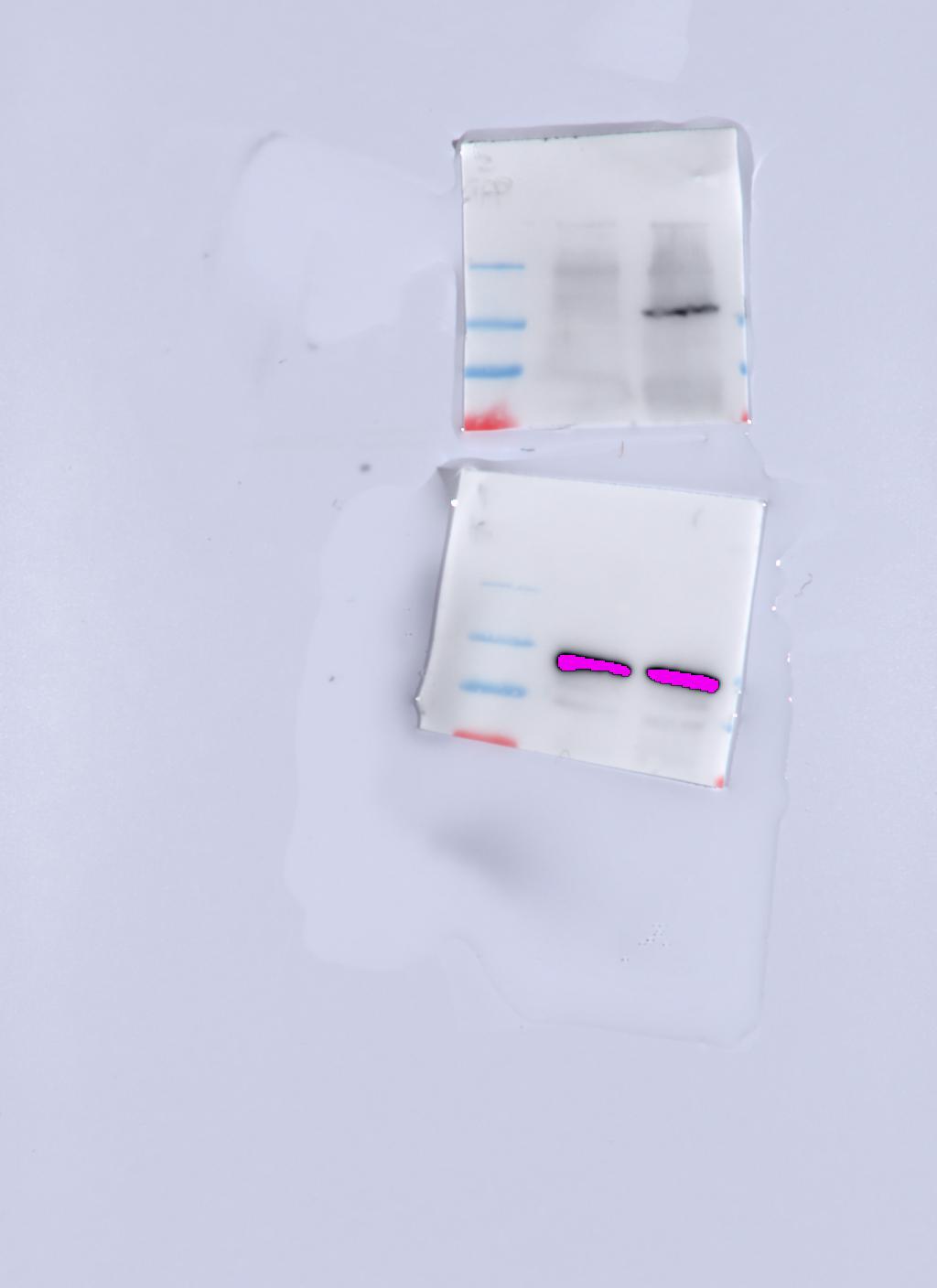

Supplement: Figure 7—source data 1. [file elife-87125-fig7-data1.zip › Figure 7-SourceData 1-2/Fig.7_Source data 2(1).jpg]

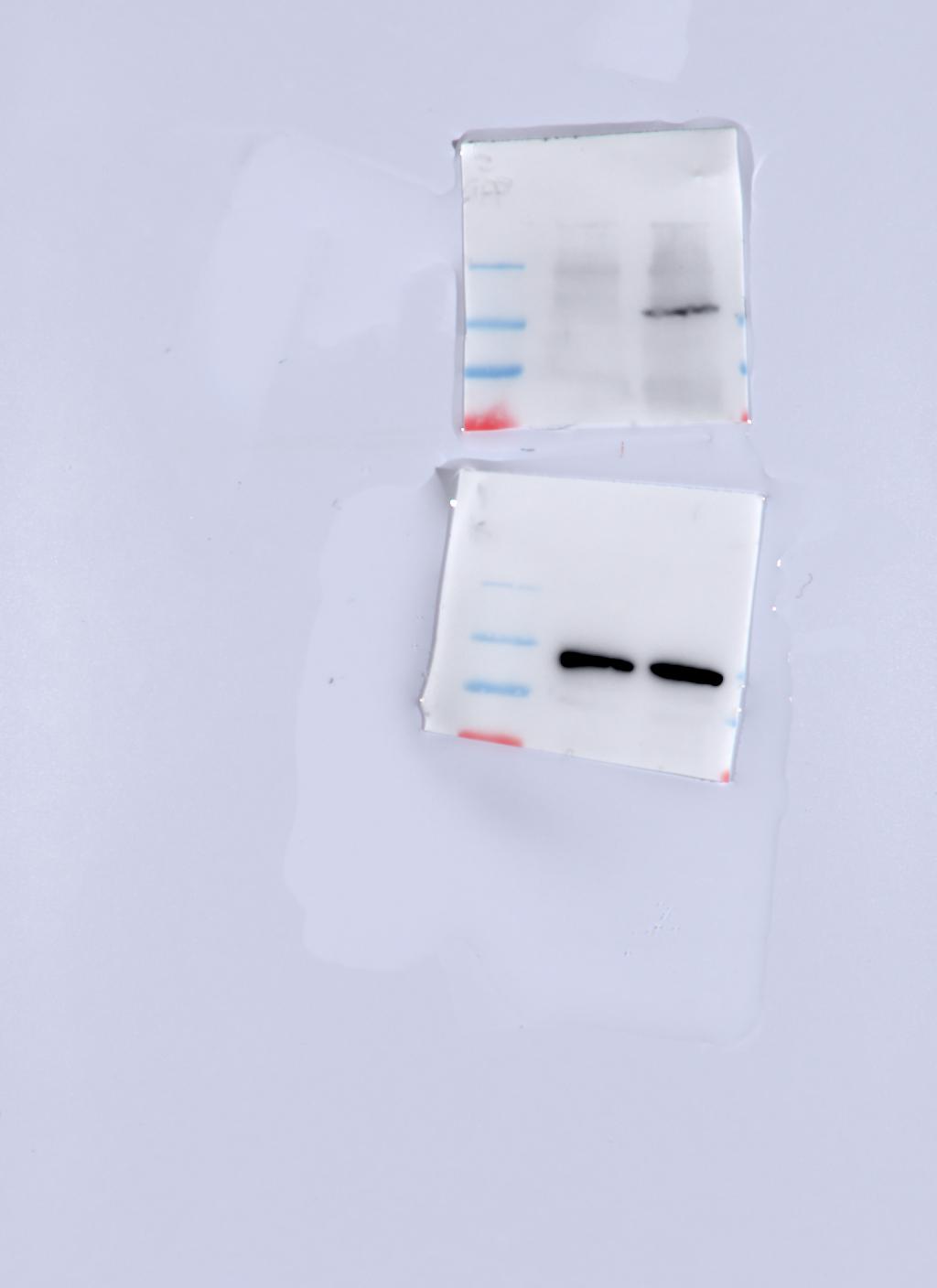

Supplement: Figure 7—source data 1. [file elife-87125-fig7-data1.zip › Figure 7-SourceData 1-2/Fig.7_Source data 2(2).jpg]

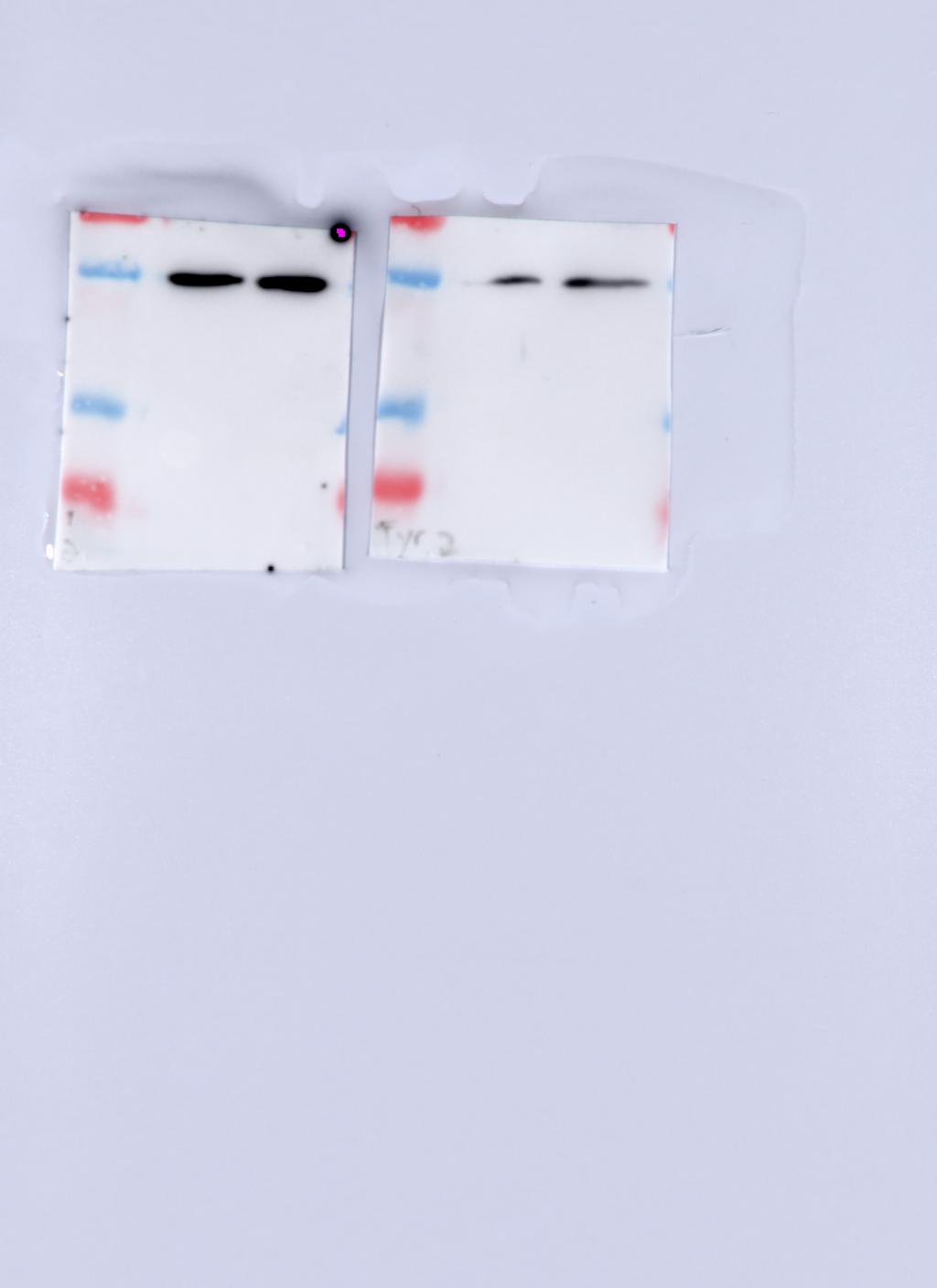

Supplement: Figure 7—source data 1. [file elife-87125-fig7-data1.zip › Figure 7-SourceData 1-2/Fig.7_Source data 2(3).jpg]

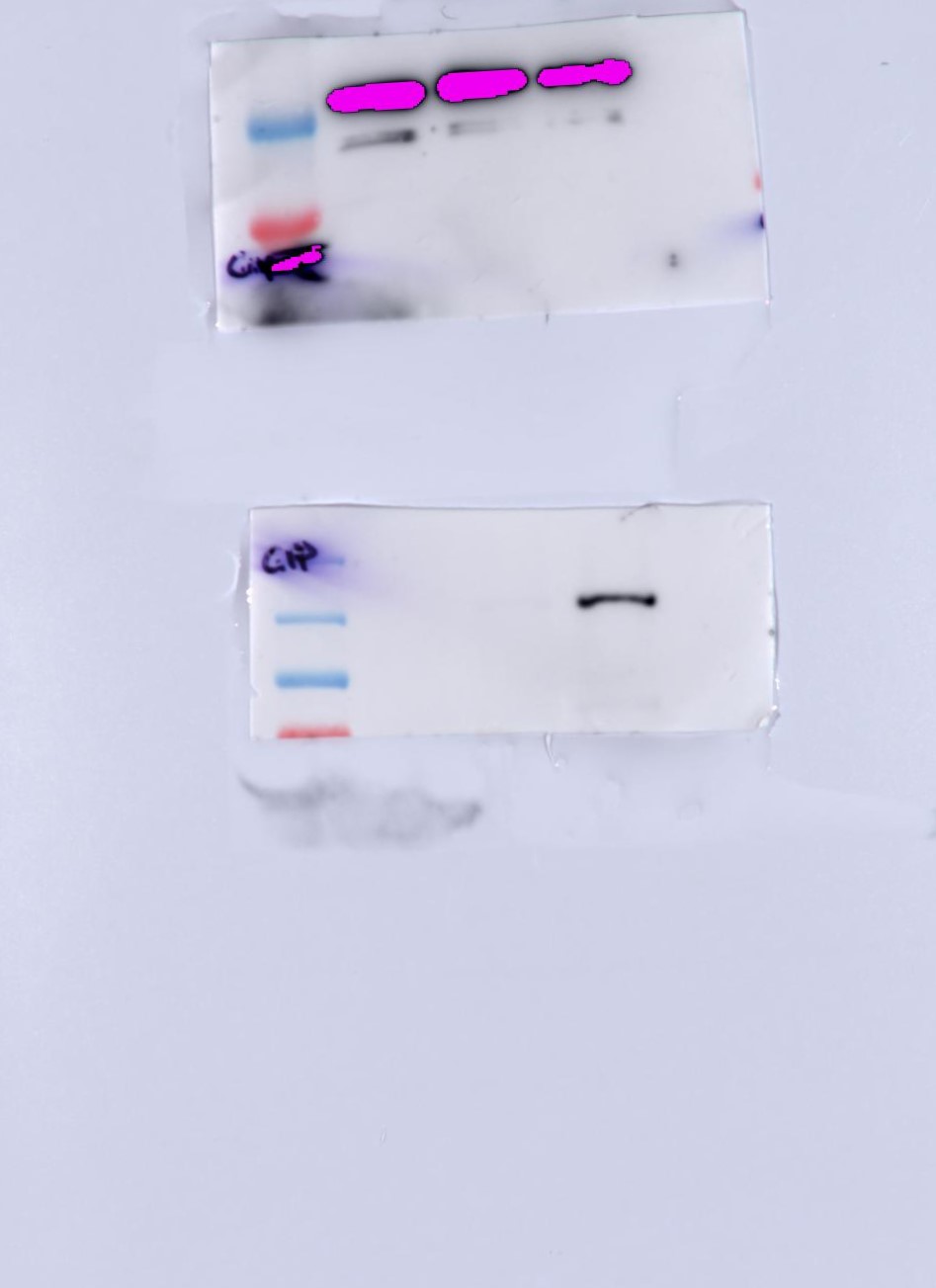

Supplement: Figure 7—source data 1. [file elife-87125-fig7-data1.zip › Figure 7-SourceData 1-2/Fig.7_Source data 1(1).jpg]

SourceDataFIG. S1

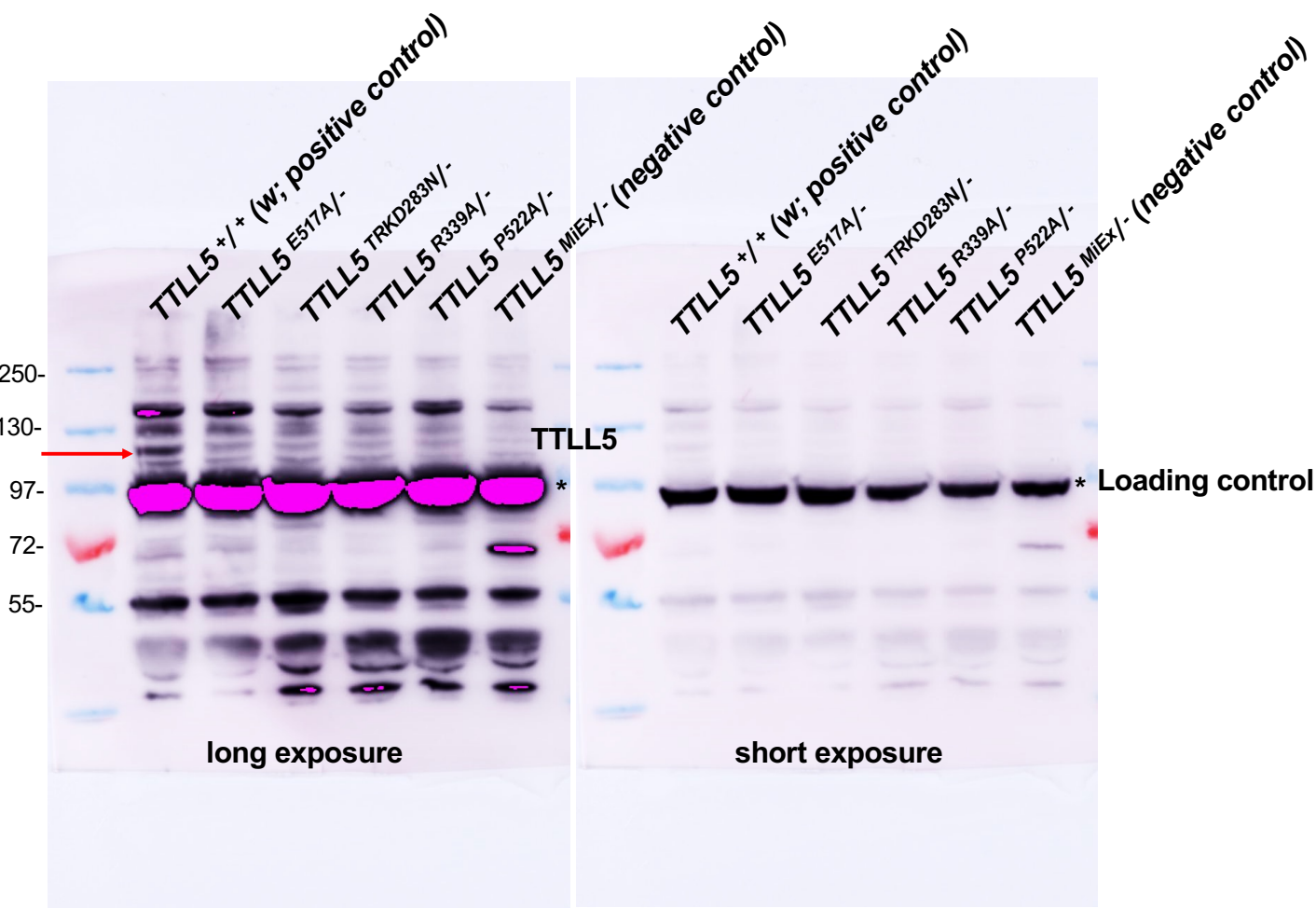

Supplement: Appendix 1—figure 1—source data 1. [file elife-87125-app1-fig1-data1.zip › Supplementary Figure 1 SourceData 1/Figure S1_SourceData 1.pdf]

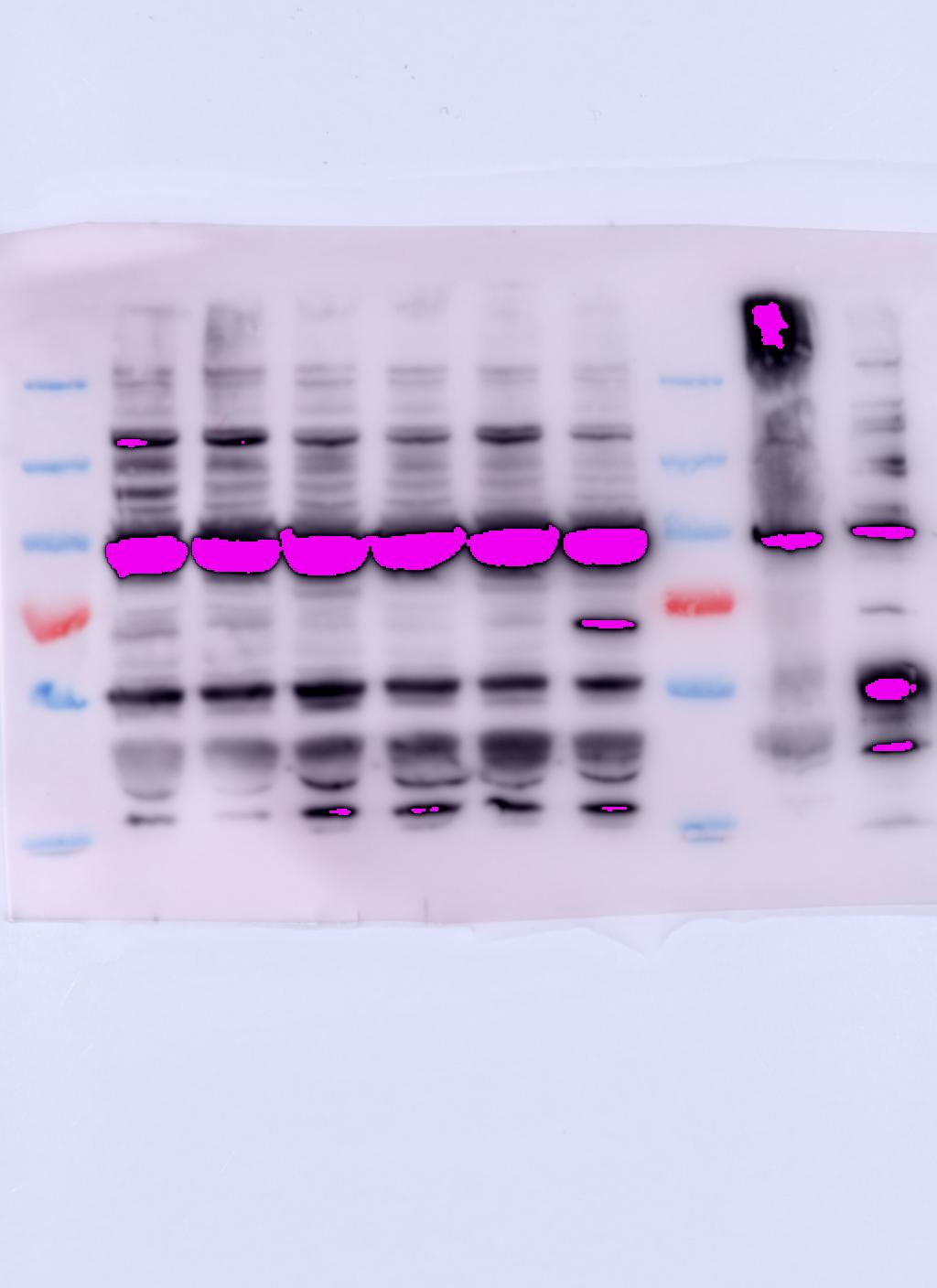

Supplement: Appendix 1—figure 1—source data 1. [file elife-87125-app1-fig1-data1.zip › Supplementary Figure 1 SourceData 1/Supplementary Figure S1_SourceData 1(1).jpg]

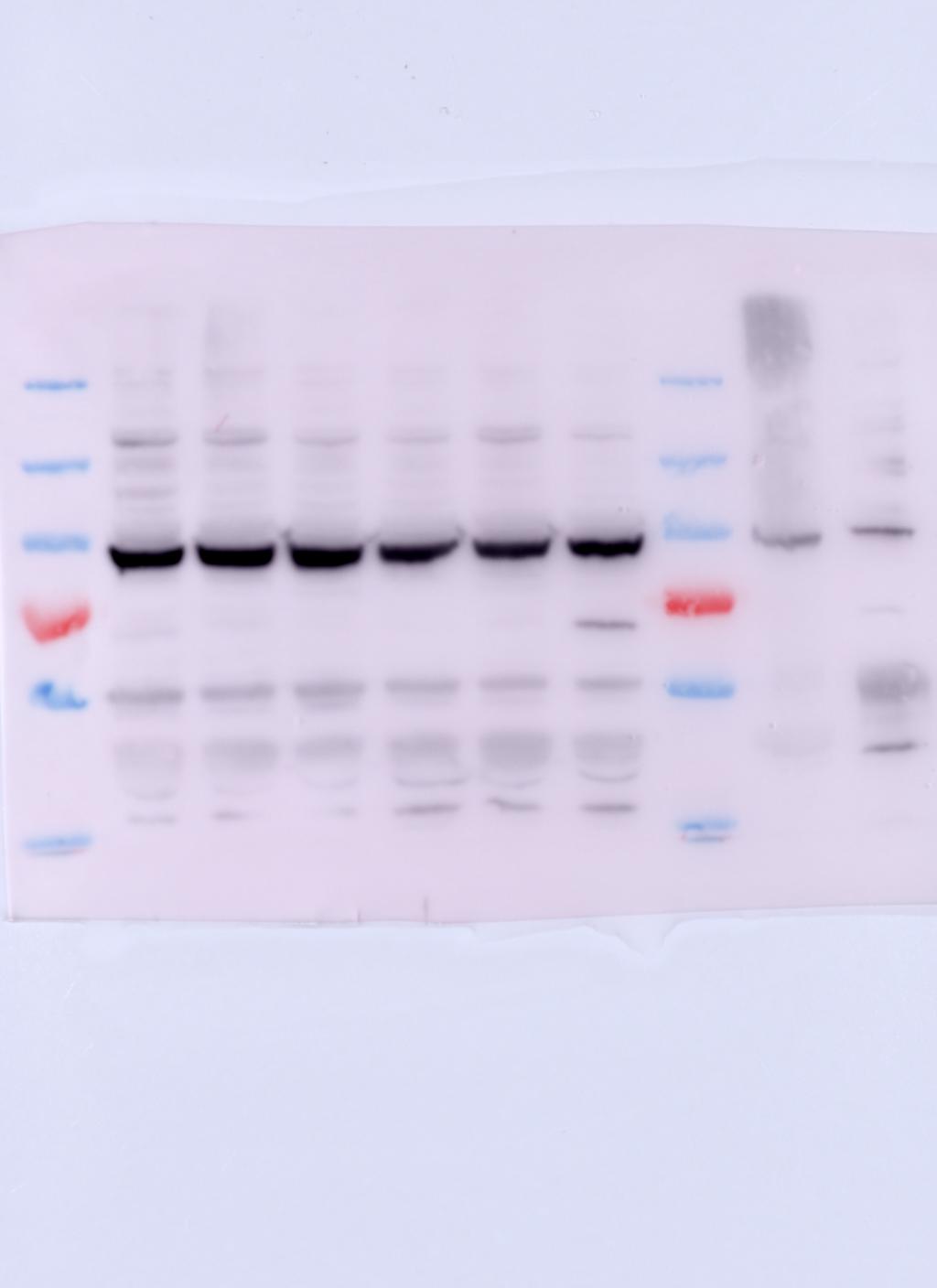

Supplement: Appendix 1—figure 1—source data 1. [file elife-87125-app1-fig1-data1.zip › Supplementary Figure 1 SourceData 1/Supplementary Figure S1_SourceData 1(2).jpg]

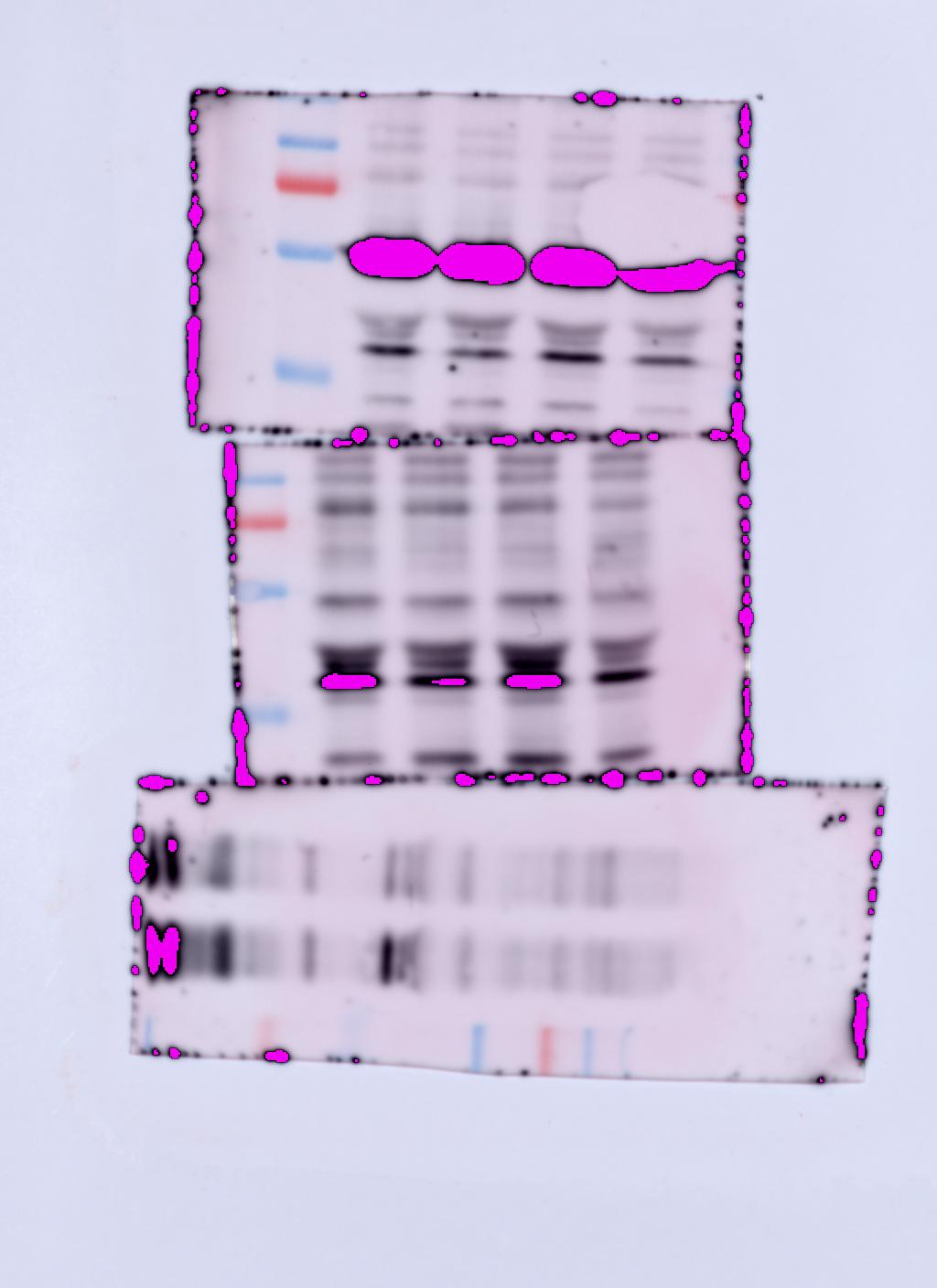

Supplement: Appendix 1—figure 2—source data 1. [file elife-87125-app1-fig2-data1.zip › Appendix 1 - Figure 2 - SourceData 1/Appendix 1 - Figure 2 - SourceData 2.jpg]

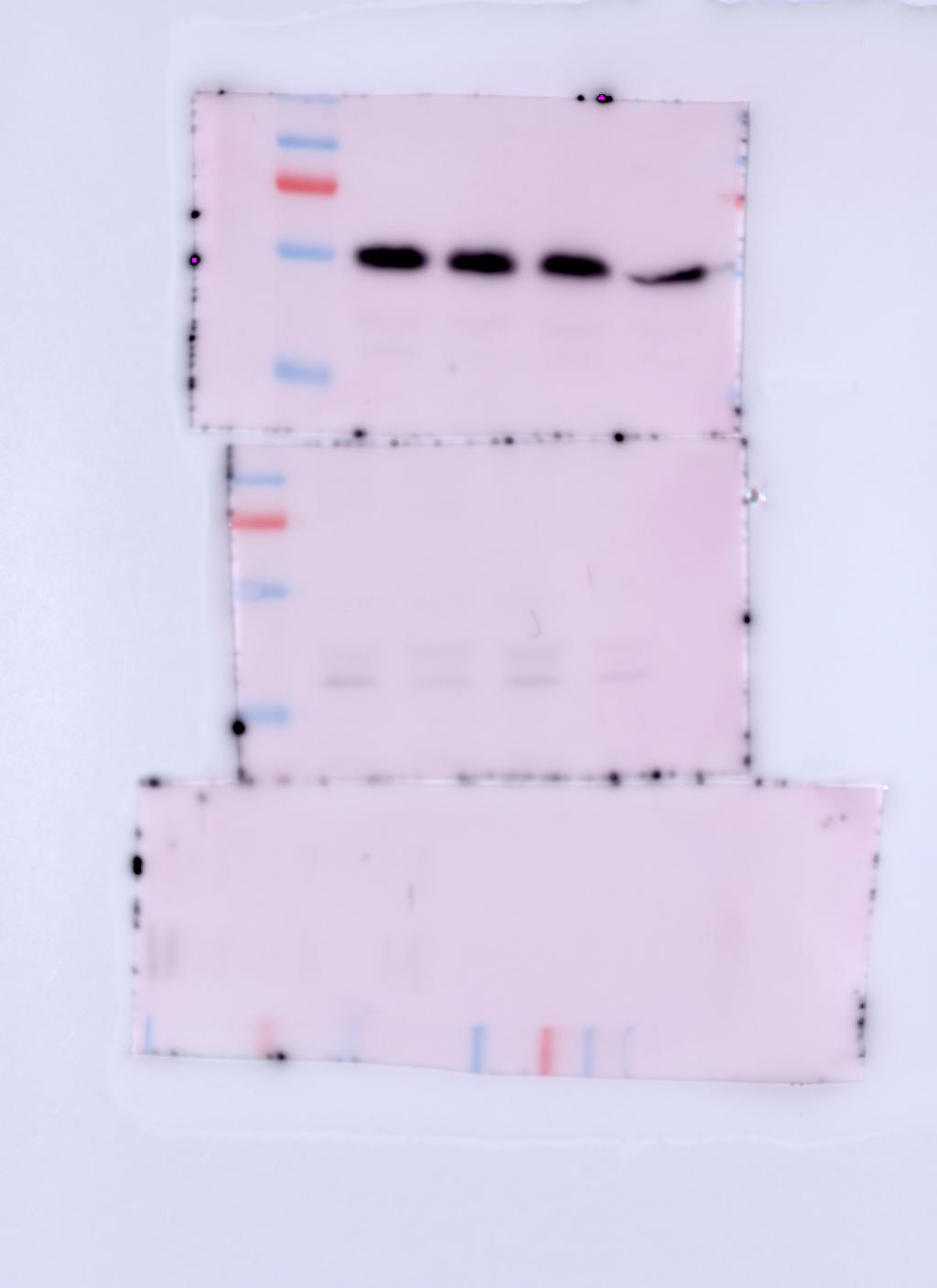

Supplement: Appendix 1—figure 2—source data 1. [file elife-87125-app1-fig2-data1.zip › Appendix 1 - Figure 2 - SourceData 1/Appendix 1 - Figure 2 - SourceData 3.jpg]

Appendix 1 - Figure 2 - SourceData 1

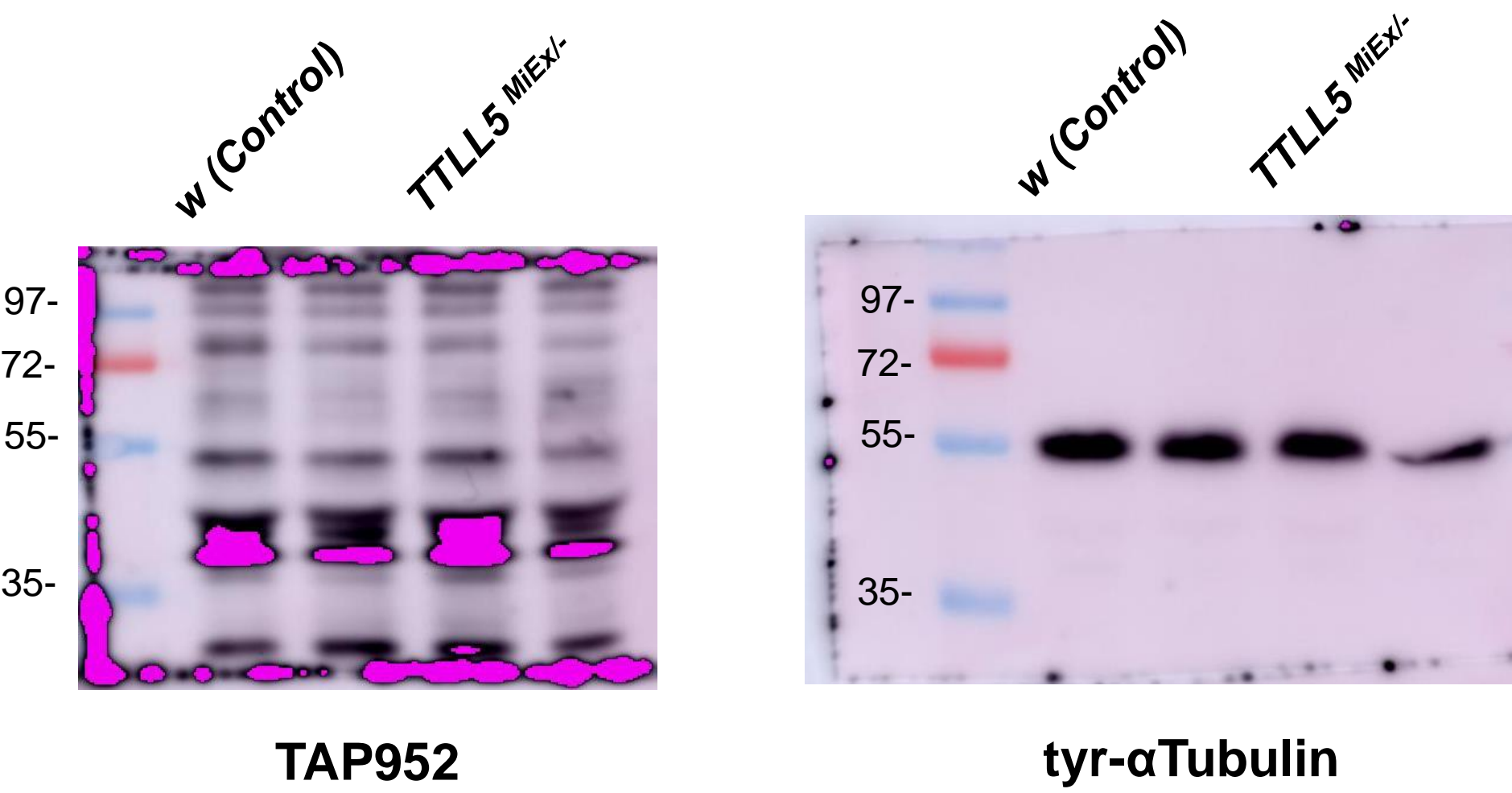

Supplement: Appendix 1—figure 2—source data 1. [file elife-87125-app1-fig2-data1.zip › Appendix 1 - Figure 2 - SourceData 1/Appendix 1 - Figure 2 - SourceData 1.pdf]

## Appendix 1 - Figure 5 - SourceData 1

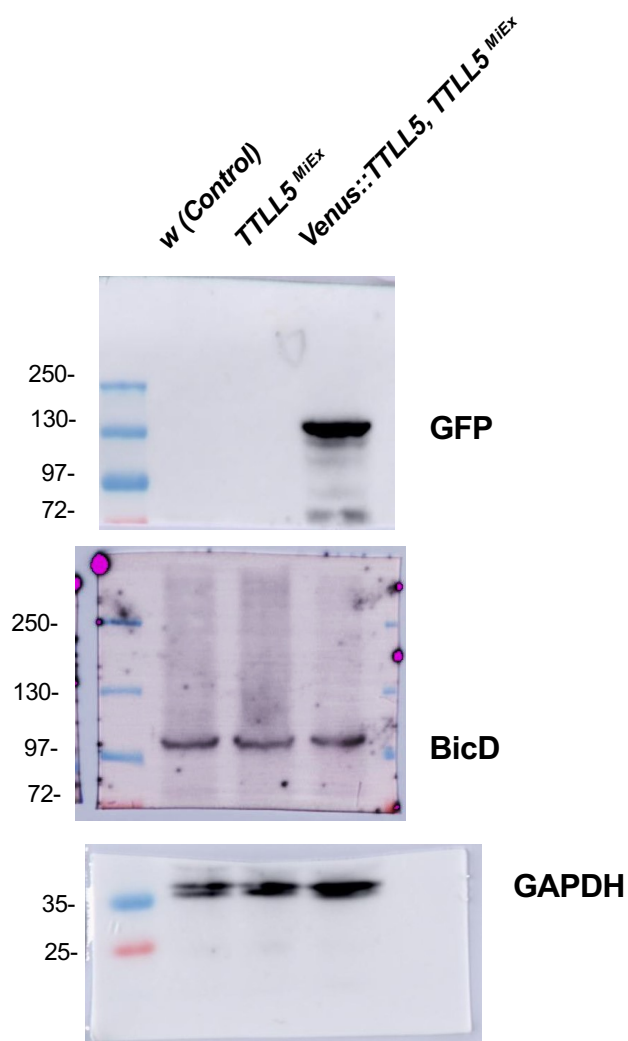

Supplement: Appendix 1—figure 5—source data 1. [file elife-87125-app1-fig5-data1.zip › Appendix 1 - Figure 5 - SourceData 1/Appendix 1 - Figure 5 - SourceData 1.pdf]

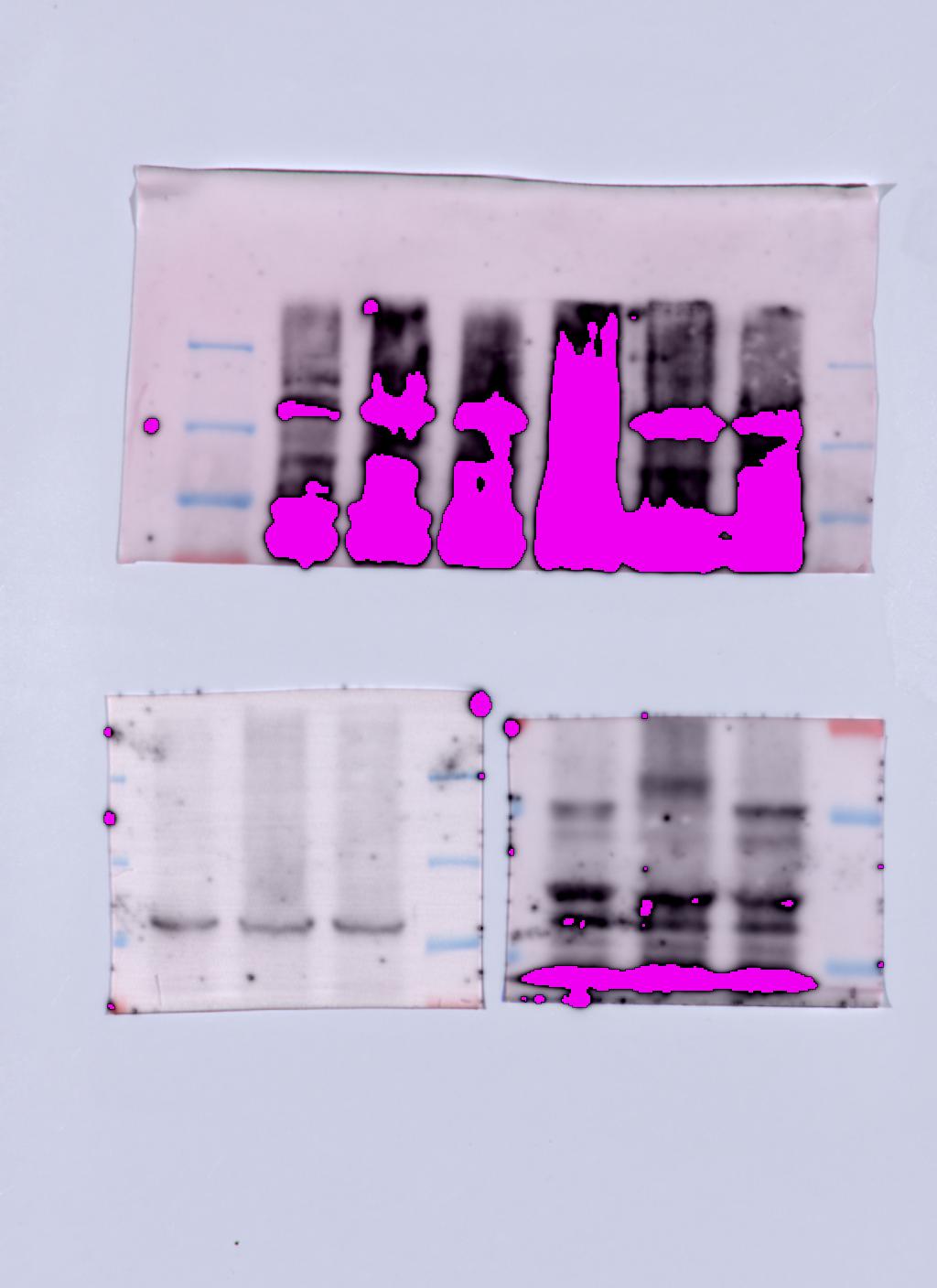

Supplement: Appendix 1—figure 5—source data 1. [file elife-87125-app1-fig5-data1.zip › Appendix 1 - Figure 5 - SourceData 1/Appendix 1 - Figure 5 - SourceData 4.jpg]
