# Supplementary material for: Differential modification of the C-terminal tails of different α-tubulins and their importance for microtubule function in vivo
Source: eLife. 2023 Jun 22;12:e87125. doi: 10.7554/eLife.87125 (PMC10335831; doi:10.7554/eLife.87125)
Supplement: Supplementary file 1. — (a) Tissue--specific expression of the 11 Drosophila TTLL genes. (b) Peptide sequence and frequency of unmodified full primary C-terminal sequence of α-tubulins identified by MS. (c) The full primary C-terminal sequence of α-tubulins modified with E sidechain modifications. The probabilities of E sidechain modifications in each peptide according to TPP are indicated for the four positions close to the C-term. (d) The full primary C-terminal sequence of α-tubulins modified by E sidechain modifications. The probabilities of E sidechain modifications in each peptide according to EasyProt, MSfragger, and PEAKS are indicated for the four positions close to the C-term. (e) Primers for cloning the sequences encoding the sgRNAs into the Drosophila transformation vector pCFD5. (f) Templates to introduce point mutations. [file elife-87125-supp1.docx]

#### Supplementary File 1 (Supplementary Tables)

**Supplementary File 1a)
Tissue specific expression of the 11 *Drosophila* *TTLL* genes**

Expression levels from high (left column) to low (right column)

| *TTLL1A* | testis | imaginal disc |  |  |  |
| --- | --- | --- | --- | --- | --- |
| *TTLL1B* | testis | imaginal disc |  |  |  |
| *TTLL3A* |  |  |  |  |  |
| *TTLL3B* | testis | imaginal disc | accessory gland | central nervous system |  |
| *TTLL4A* | testis | imaginal disc | ovary | central nervous system |  |
| *TTLL4B* | testis | imaginal disc | accessory gland | fat body |  |
| *TTLL5* | ovary | imaginal disc | testis | accessory gland | central nervous system |
| *TTLL6A* | testis | imaginal disc | accessory gland |  |  |
| *TTLL6B* | testis | imaginal disc | accessory gland | fat body |  |
| *TTLL12* | central nervous system | carcass | ovary/head/  imaginal disc | fat body |  |
| *TTLL15* |  |  |  |  |  |

data from FlyBase (https://flybase.org)

**Supplementary File 1b)
The unmodified full primary C-terminal sequence of α-tubulins by MS**

TPP (Trans-Proteomics Pipeline) searches against the uniprotKB Drosophila database identified the following C-terminal fragments of
**Tub84B/αTub84D**

KDYEEVGMDSGDGEGEGAEEY

REDLAALEKDYEEVGMDSGDGEGEGAEEY

**αTub67C:**

RDFEEVGLDNAEEGGDEDFDEF

RENIAVLERDFEEVGLDNAEEGGDEDFDEF

| Genotype | C-terminal peptide | # PSM/peptide |
| --- | --- | --- |
| *w* | KDYEEVGMDSGDGEGEGAEEY | 6 |
| *w* | REDLAALEKDYEEVGMDSGDGEGEGAEEY | 11 |
| *w* | RDFEEVGLDNAEEGGDEDFDEF | 8 |
| *w* | RENIAVLERDFEEVGLDNAEEGGDEDFDEF | 4 |
| *TTLL5^MiEx/-^* | KDYEEVGMDSGDGEGEGAEEY | 2 |
| *TTLL5^MiEx/-^* | REDLAALEKDYEEVGMDSGDGEGEGAEEY | 4 |
| *TTLL5^MiEx/-^* | RDFEEVGLDNAEEGGDEDFDEF | 2 |
| *TTLL5^MiEx/-^* | RENIAVLERDFEEVGLDNAEEGGDEDFDEF | 2 |
| *TTLL5^pBac/-^* | KDYEEVGMDSGDGEGEGAEEY | 11 |
| *TTLL5^pBac/-^* | REDLAALEKDYEEVGMDSGDGEGEGAEEY | 12 |
| *TTLL5^pBac/-^* | RDFEEVGLDNAEEGGDEDFDEF | 8 |
| *TTLL5^pBac/-^* | RENIAVLERDFEEVGLDNAEEGGDEDFDEF | 4 |

**Supplementary File 1c)**

**The full primary C-terminal sequence of α-tubulins modified with Glu sidechain modifications**

The peptides identified by TPP

| Genotype | E numbers and potential postions on C-terminal peptide evaluated by pipeline | The probability of E modification on different residues |
| --- | --- | --- |
| *w* | KDYEEVGMDSGDGE[E1]GEGAEEY | KDYE(0.000)E(0.000)VGMDSGDGE(0.263)GE(0.263)GAE(0.263)E(0.211)Y |
| *w* | KDYEEVGMDSGDGE[E1]GE[E1]GAEEY | KDYE(0.001)E(0.000)VGMDSGDGE(0.500)GE(0.500)GAE(0.500)E(0.499)Y |
| *w* | KDYEEVGMDSGDGEGEGAE[E1]EY | KDYE(0.000)E(0.000)VGMDSGDGE(0.000)GE(0.001)GAE(0.499)E(0.499)Y |
| *w* | KDYEEVGMDSGDGEGE[E1]GAEEY | KDYE(0.000)E(0.000)VGMDSGDGE(0.160)GE(0.779)GAE(0.060)E(0.000)Y |
| *w* | KDYEEVGMDSGDGEGEGAE[E1]EY | KDYE(0.000)E(0.000)VGMDSGDGE(0.000)GE(0.002)GAE(0.526)E(0.472)Y |
| *w* | KDYEEVGMDSGDGEGE[E1]GAEEY | KDYE(0.000)E(0.000)VGMDSGDGE(0.031)GE(0.968)GAE(0.000)E(0.000)Y |
| *w* | KDYEEVGMDSGDGE[E1]GE[E1]GAEEY | KDYE(0.006)E(0.000)VGMDSGDGE(0.502)GE(0.502)GAE(0.502)E(0.489)Y |
| *w* | KDYEEVGMDSGDGE[E1]GE[E1]GAEEY | KDYE(0.000)E(0.000)VGMDSGDGE(0.500)GE(0.500)GAE(0.500)E(0.500)Y |
| *w* | REDLAALEKDYEEVGMDSGDGE[E1]GE[E1]GAE[E1]EY | RE(0.000)DLAALE(0.000)KDYE(0.000)E(0.000)VGMDSGDGE(0.751)GE(0.751)GAE(0.749)E(0.749)Y |
| *w* | REDLAALEKDYEEVGMDSGDGE[E1]GE[E1]GAEEY | RE(0.000)DLAALE(0.000)KDYE(0.078)E(0.078)VGMDSGDGE(0.624)GE(0.624)GAE(0.299)E(0.299)Y |
| *w* | REDLAALEKDYEEVGMDSGDGE[E1]GE[E1]GAEEY | RE(0.000)DLAALE(0.000)KDYE(0.002)E(0.002)VGMDSGDGE(0.566)GE(0.566)GAE(0.566)E(0.297)Y |
| *w* | REDLAALEKDYEEVGMDSGDGE[E1]GEGAEEY | RE(0.000)DLAALE(0.000)KDYE(0.003)E(0.003)VGMDSGDGE(0.249)GE(0.249)GAE(0.249)E(0.249)Y |
| *w* | REDLAALEKDYEEVGMDSGDGE[E1]GE[E1]GAEEY | RE(0.000)DLAALE(0.000)KDYE(0.001)E(0.001)VGMDSGDGE(0.510)GE(0.510)GAE(0.510)E(0.466)Y |
| *w* | REDLAALEKDYEEVGMDSGDGEGE[E1]GAEEY | RE(0.000)DLAALE(0.000)KDYE(0.000)E(0.000)VGMDSGDGE(0.253)GE(0.371)GAE(0.371)E(0.005)Y |
| *w* | REDLAALEKDYEEVGMDSGDGE[E1]GEGAEEY | RE(0.000)DLAALE(0.000)KDYE(0.000)E(0.000)VGMDSGDGE(0.250)GE(0.250)GAE(0.250)E(0.250)Y |
| *w* | REDLAALEKDYEEVGMDSGDGE[E1]GEGAEEY | RE(0.000)DLAALE(0.000)KDYE(0.001)E(0.001)VGMDSGDGE(0.250)GE(0.250)GAE(0.250)E(0.250)Y |
| *w* | REDLAALEKDYEEVGMDSGDGE[E1]GE[E1]GAE[E1]EY | RE(0.000)DLAALE(0.000)KDYE(0.000)E(0.000)VGMDSGDGE(0.756)GE(0.756)GAE(0.756)E(0.731)Y |
| *w* | REDLAALEKDYEEVGMDSGDGEGE[E1]GAEEY | RE(0.000)DLAALE(0.000)KDYE(0.000)E(0.000)VGMDSGDGE(0.000)GE(1.000)GAE(0.000)E(0.000)Y |
| *w* | REDLAALEKDYEEVGMDSGDGE[E1]GE[E1]GAEEY | RE(0.000)DLAALE(0.000)KDYE(0.000)E(0.000)VGMDSGDGE(0.996)GE(0.996)GAE(0.008)E(0.000)Y |
| *w* | REDLAALEKDYEEVGMDSGDGE[E1]GE[E1]GAE[E1]EY | RE(0.000)DLAALE(0.000)KDYE(0.000)E(0.000)VGMDSGDGE(0.751)GE(0.751)GAE(0.749)E(0.749)Y |
| *w* | REDLAALEKDYEEVGMDSGDGEGE[E1]GAEEY | RE(0.000)DLAALE(0.000)KDYE(0.000)E(0.000)VGMDSGDGE(0.000)GE(0.786)GAE(0.213)E(0.001)Y |
| *w* | REDLAALEKDYEEVGMDSGDGE[E1]GE[E1]GAEEY | RE(0.000)DLAALE(0.000)KDYE(0.000)E(0.000)VGMDSGDGE(0.500)GE(0.500)GAE(0.500)E(0.500)Y |
| *w* | REDLAALEKDYEEVGMDSGDGEGE[E1]GAEEY | RE(0.000)DLAALE(0.000)KDYE(0.000)E(0.000)VGMDSGDGE(0.001)GE(0.333)GAE(0.333)E(0.333)Y |
| *w* | REDLAALEKDYEEVGMDSGDGE[E1]GE[E1]GAE[E1]EY | RE(0.000)DLAALE(0.000)KDYE(0.023)E(0.023)VGMDSGDGE(0.758)GE(0.758)GAE(0.758)E(0.680)Y |
| *w* | REDLAALEKDYEEVGMDSGDGEGE[E1]GAEEY | RE(0.000)DLAALE(0.000)KDYE(0.000)E(0.000)VGMDSGDGE(0.000)GE(1.000)GAE(0.000)E(0.000)Y |
| *w* | REDLAALEKDYEEVGMDSGDGE[E1]GEGAE[E1]EY | RE(0.000)DLAALE(0.000)KDYE(0.000)E(0.000)VGMDSGDGE(0.894)GE(0.210)GAE(0.889)E(0.006)Y |
| *w* | REDLAALEKDYEEVGMDSGDGE[E1]GEGAEEY | RE(0.000)DLAALE(0.000)KDYE(0.000)E(0.000)VGMDSGDGE(0.257)GE(0.257)GAE(0.257)E(0.228)Y |
| *w* | REDLAALEKDYEEVGMDSGDGEGE[E1]GAEEY | RE(0.000)DLAALE(0.000)KDYE(0.000)E(0.000)VGMDSGDGE(0.000)GE(0.997)GAE(0.003)E(0.000)Y |
| *w* | REDLAALEKDYEEVGMDSGDGE[E1]GEGAEEY | RE(0.000)DLAALE(0.000)KDYE(0.000)E(0.000)VGMDSGDGE(0.371)GE(0.371)GAE(0.255)E(0.002)Y |
| *w* | KDYEEVGMDSGDGE[E2]GEGAEEY | KDYE(0.000)E(0.000)VGMDSGDGE(0.250)GE(0.250)GAE(0.250)E(0.250)Y |
| *w* | REDLAALEKDYEEVGMDSGDGE[E2]GEGAEEY | RE(0.000)DLAALE(0.000)KDYE(0.001)E(0.001)VGMDSGDGE(0.255)GE(0.255)GAE(0.255)E(0.233)Y |
| *w* | REDLAALEKDYEEVGMDSGDGE[E2]GEGAEEY | RE(0.000)DLAALE(0.000)KDYE(0.001)E(0.001)VGMDSGDGE(0.284)GE(0.284)GAE(0.284)E(0.145)Y |
| *w* | REDLAALEKDYEEVGMDSGDGE[E2]GEGAEEY | RE(0.000)DLAALE(0.000)KDYE(0.000)E(0.000)VGMDSGDGE(0.690)GE(0.309)GAE(0.000)E(0.002)Y |
| *w* | REDLAALEKDYEEVGMDSGDGEGE[E2]GAEEY | RE(0.000)DLAALE(0.000)KDYE(0.000)E(0.000)VGMDSGDGE(0.123)GE(0.563)GAE(0.314)E(0.000)Y |
| *w* | REDLAALEKDYEEVGMDSGDGE[E2]GEGAEEY | RE(0.000)DLAALE(0.000)KDYE(0.037)E(0.037)VGMDSGDGE(0.315)GE(0.315)GAE(0.148)E(0.148)Y |
| *w* | REDLAALEKDYEEVGMDSGDGE[E2]GEGAEEY | RE(0.000)DLAALE(0.000)KDYE(0.000)E(0.000)VGMDSGDGE(0.250)GE(0.250)GAE(0.250)E(0.250)Y |
| *w* | REDLAALEKDYEEVGMDSGDGE[E2]GEGAEEY | RE(0.000)DLAALE(0.000)KDYE(0.140)E(0.140)VGMDSGDGE(0.180)GE(0.180)GAE(0.180)E(0.180)Y |
| *w* | REDLAALEKDYEEVGMDSGDGE[E3]GEGAEEY | RE(0.000)DLAALE(0.000)KDYE(0.000)E(0.000)VGMDSGDGE(0.252)GE(0.252)GAE(0.252)E(0.243)Y |
| *w* | REDLAALEKDYEEVGMDSGDGE[E3]GEGAEEY | RE(0.000)DLAALE(0.000)KDYE(0.000)E(0.000)VGMDSGDGE(0.250)GE(0.250)GAE(0.250)E(0.250)Y |
| *w* | REDLAALEKDYEEVGMDSGDGE[E3]GEGAEEY | RE(0.000)DLAALE(0.000)KDYE(0.001)E(0.001)VGMDSGDGE(0.257)GE(0.257)GAE(0.257)E(0.229)Y |
| *w* | REDLAALEKDYEEVGMDSGDGE[E3]GEGAEEY | RE(0.000)DLAALE(0.000)KDYE(0.000)E(0.000)VGMDSGDGE(0.250)GE(0.250)GAE(0.250)E(0.250)Y |
| *TTLL5^pBac/-^* | REDLAALEKDYEEVGMDSGDGE[E1]GEGAEEY | RE(0.000)DLAALE(0.000)KDYE(0.023)E(0.001)VGMDSGDGE(0.244)GE(0.244)GAE(0.244)E(0.244)Y |

**Supplementary File 1d)**

**The full primary C-terminal sequence of α-tubulins modified by E**

The peptides were analyzed by EasyProt, msfragger, and PEAKS programs.

[+129.04] indicates1E, [+258.09] indicates 2E and [+387.13] indicates 3E

| Genotype | Programs | E numbers and potential postions on C-terminal peptide |
| --- | --- | --- |
| *w* | EasyProt | A.REDLAALEKDYE[+129.04]EVGMDSGDGEGEGAEE[+258.09]Y.- |
| *w* | EasyProt | A.REDLAALEKDYEEVGMDSGDGEGE[+129.04]GAEEY.- |
| *w* | EasyProt | A.REDLAALEKDYEEVGMDSGDGE[+129.04]GEGAEEY.- |
| *w* | EasyProt | A.REDLAALEKDYEEVGMDSGDGEGE[+129.04]GAEEY.- |
| *w* | EasyProt | A.REDLAALEKDYEEVGMDSGDGEGEGAE[+129.04]E[+258.09]Y.- |
| *w* | EasyProt | A.REDLAALEKDYEEVGMDSGDGEGEGAE[+258.09]EY.- |
| *w* | EasyProt | A.REDLAALEKDYEEVGMDSGDGEGEGAE[+387.13]EY.- |
| *w* | EasyProt | E.KDYEEVGMDSGDGEGE[+129.04]GAEEY.- |
| *w* | msfragger | E.KDYEEVGMDSGDGE[+129.04]GEGAEEY.- |
| *w* | msfragger | E.KDYEEVGMDSGDGEGE[+129.04]GAEEY.- |
| *w* | PEAKS | A.REDLAALEKDYEEVGMDSGDGE[+258.09]GEGAEEY |
| *w* | PEAKS | A.REDLAALEKDYEEVGMDSGDGEGE[+258.09]GAEEY |
| *w* | PEAKS | A.REDLAALEKDYEEVGMDSGDGE[+129.04]GEGAE[+129.04]EY |
| *w* | PEAKS | A.REDLAALEKDYEEVGMDSGDGE[+129.04]GEGAEE[+258.09]Y |
| *w* | PEAKS | A.REDLAALEKDYEEVGMDSGDGE[+129.04]GEGAEEY |
| *w* | PEAKS | A.REDLAALEKDYEEVGMDSGDGE[+258.09]GEGAEEY |
| *w* | PEAKS | A.REDLAALEKDYEEVGMDSGDGEGE[+129.04]GAEEY |
| *w* | PEAKS | A.REDLAALEKDYEEVGMDSGDGEGEGAE[+258.09]EY |
| *w* | PEAKS | E.KDYEEVGMDSGDGE[+129.04]GEGAEEY |
| *w* | PEAKS | E.KDYEEVGMDSGDGE[+258.09]GEGAEEY |
| *w* | PEAKS | E.KDYEEVGMDSGDGEGE[+129.04]GAEEY |

#### Supplementary File 1e)

#### Primers for cloning the sequences encoding the sgRNAs into the *Drosophila* transformation vector pCFD5

| TTLL5 R339 top ^5'^ TGC AGC GGC ATC CAG TCG AGG AAG |
| --- |
| TTLL5 R339 rev ^5'^ AAA CCT TCC TCG ACT GGA TGC CGC |
| TTLL5 K282 top ^5'^ TGC ATC CTA CGA AAT GAC ACG AA |
| TTLL5 K282 rev ^5'^ AAA CTT CGT GTC ATT TCG TAG GA |
| TTLL5 E517/P522 top ^5'^ TGC AAG ATT GAT TTC CAG TAG CCA |
| TTLL5 E517/P522 rev ^5'^ AAA CTG GCT ACT GGA AAT CAA TCT |

**Supplementary File 1f)
Templates to introduce point mutations**

| ***TTLL5^R339A/-^*** |
| --- |
| = Arg (AGG) -> Ala (**GCT**) on the reverse strand (AGC); introducing an AluI site (AGCT) at the same time; PAM (underlined) on the other strand (5'TGG) |
| ^5'^GAATTATAGCAAGTTGTTGGTTTAGTTACCGAATTAACAATAAATATTCC**AGC**T'CCTCGACTGGATGCCGCTGGCTTAACTATCCATGGTCCGCGATGCTTGTT |
| ***TTLL5^E517A/-^*** |
| = Glu (GAA) -> Ala (**GCC**) on the reverse strand (GGC); introducing a HaeIII site (GGCC) at the same time; PAM (underlined) on this strand (5'TGG) ^5'^ACCTTAGTGTCCAAAGGACTATCCACACCCATTGACGGCGATAGATTGAT**GGC**CAGTAG'CCATGGCTTAAGTGCGTTGTCTATTAATATATCGAATCCATACAGCTCAA |
| ***TTLL5^P522A/-^*** |
| = Pro (CCG) -> Ala (GCG) on the reverse strand (CGC); introducing a BsaHI site (GRCGYC) at the same time; PAM (underlined) on this strand (5'TGG); grey nucleotide changes that are silent mutations to prevent multiple recognition after DNA was cut |
| ^5'^GTCTGCCATCAGACATGACTTAACCTTAGTGTCCAAAGGACTATCCACACCCATTGA**CGC**CGATAGATTGATTTC**G**AG**A**AG'CCATGGCTTAAGTGCGTTGTCTATTAATATATCGAATCCATACAGCTCAA |
